# Supplementary material for: Hydrophobized Reversed-Phase Adsorbent for Protection of Dairy Cattle against Lipophilic Toxins from Diet. Efficiensy In Vitro and In Vivo
Source: Toxins (Basel). 2019 May 7;11(5):256. doi: 10.3390/toxins11050256 (PMC6563209; doi:10.3390/toxins11050256)
Supplement: Supplementary file 1 [file toxins-11-00256-s001.pdf]

Supplementary Materials: Hydrophobized Reversed-Phase Adsorbent for Protection of Dairy Cattle against Lipophilic Toxins from Diet. Efficiensy in Vitro and in Vivo

Alexander Sotnichenko, Evgeny Pantsov, Dmitry Shinkarev and Victor Okhanov

Table S1. Calculated partition coefficients of some fungal and bacterial metabolites*.

No.	Metabolite	Molecular Formula	Molecular Weight	XLog P3-AA
1.	Aspergillomarasmin	C9H14N2O8	278.2	-10.0
2.	Ustiloxin B	C26H39N5O12S	645.6	-7.8
3.	Lycomarasmin	C9H15N3O7	277.2	-7.5
4.	Adenophostin A	C16H20N5Na6O18P3	801.2	-7.0
5.	Ustiloxin A	C28H43N5O12S	673.7	-6.8
6.	Adenophostin B	C18H28N5O19P3	711.3	-6.5
7.	Ustiloxin C	C23H34N4O10S	558.6	-4.6
8.	Cephalosporin C	C16H21N3O7S	415.4	-4.4
9.	Paracelsin	C88H145N23O24	1909.2	-3.9
10.	Alterporriol E	C32H30O16	670.5	-3.0
11.	Ustiloxin F	C21H30N4O8	466.4	-3.0
12.	Epicorazine C	C18H18N2O7S2	438.4	-2.7
13.	Vancomycin	C66H75Cl2N9O24	1449.2	-2.6
14.	Cephalosporin N	C14H21N3O6S	359.3	-2.5
15.	Deoxynivalenol-3-glucoside	C21H30O11	458.4	-2.3
16.	Ustiloxin D	C23H34N4O8	494.5	-2.0
17.	Altersolanol D	C16H16O8	336.2	-1.9
18.	Altersolanol F	C32H30O15	654.5	-1.9
19.	Alterporriol F	C32H30O15	654.5	-1.9
20.	Mizoribine	C9H13N3O6	259.2	-1.9
21.	Nivalenol	C15H20O7	312.3	-1.9
22.	Alamethicin	C92H150N22O25	1964.3	-1.8
23.	Epicorazine A	C18H16N2O6S2	420.4	-1.7
24.	Epicorazine B	C18H16N2O6S2	420.4	-1.7
25.	6-ile-Ustiloxin	C24H36N4O8	508.5	-1.7
26.	Ampelanol	C16H20O8	340.3	-1.5
27.	Rugulosin C	C30H22O12	574.4	-1.5
28.	Altersolanol A	C16H16O8	336.2	-1.3

29.	Altersolanol E	C16H16O8	336.2	-1.3
30.	Sphingofungin E	C21H39NO7	417.5	-1.3
31.	*Swainsonine*	C8H15NO3	173.2	-1.3
32.	Austdiol	C12H12O5	236.2	-1.2
33.	Cyclopiamide J	C22H24N2O7	428.4	-1.2
34.	Phomoeuphorbin C	C15H20O6	296.3	-1.2
35.	Phomoeuphorbin D	C15H20O6	296.3	-1.2
36.	Aspinonene	C9H16O4	188.2	-1.1
37.	Fusarenon X	C17H22O8	354.3	-1.1
38.	Patulin	C7H6O4	154.1	-1.0
39.	Scirpentriol	C15H22O5	282.3	-1.0
40.	Fumonisin C1	C33H57NO15	707.8	-0.9
41.	Kojic acid	C6H6O4	142.1	-0.9
42.	Altersolanol Q	C16H20O6	308.3	-0.9
43.	Altersolanol N	C18H18O9	378.3	-0.8
44.	Decarestrictine D	C10H16O5	216.2	-0.8
45.	Decarestrictine O	C10H16O5	216.2	-0.8
46.	Pseurotin E	C22H23NO9	445.4	-0.8
47.	15-Acetyldeoxynivalenol	C17H22O7	338.3	-0.7
48.	Deoxynivalenol (DON)	C15H20O6	296.3	-0.7
49.	Dihydroaltersolanol A	C16H18O7	322.3	-0.7
50.	Gliotoxin	C13H14N2O4S2	326.3	-0.7
51.	Butenolide	C4H4O2	84.0	-0.6
52.	3-Nitropropionic acid	C11H12ClNO4	257.6	-0.6
53.	Puberulic acid	C8H6O6	198.1	-0.6
54.	Pyrenocine K	C12H14O5	238.2	-0.6
55.	Terrein	C8H10O3	154.1	-0.6
56.	15-Acetyldeoxynivalenol	C17H22O7	338.3	-0.5
57.	Altenin	C9H14O6	218.2	-0.5
58.	Fumonisin B1	C34H59NO15	721.8	-0.5
59.	Puberulonic acid	C9H4O7	224.1	-0.5
60.	Arabenoic acid	C6H10O4	146.1	-0.4
61.	Aspergillide E	C25H30N4O5	466.5	-0.4
62.	Eremofortin E	C17H21NO6	335.3	-0.4
63.	Monoacetoxyscirpenol	C17H24O6	324.3	-0.4
64.	Mytomycin	C15H18N4O5	334.3	-0.4
65.	Phomopsin B	C36H46N6O12	754.7	-0.4

66.	Phomoeuphorbin A	C15H16O6	292.2	-0.4
67.	Aflatoxin-M1-8,9-epoxide	C17H12O8	344.2	-0.3
68.	Aflatoxin M2A	C17H14O8	346.2	-0.3
69.	Decarestrictine B	C10H14O5	214.2	-0.3
70.	Depudecin	C11H16O4	212.2	-0.3
71.	Neosolaniol	C19H26O8	382.4	-0.3
72.	Rugulosin B	C30H22O11	558.4	-0.3
73.	Aflatoxin B1 diol	C17H14O8	346.3	-0.2
74.	Altersolanol C	C16H16O7	320.2	-0.2
75.	Botryodiplodin	C7H12O3	144.1	-0.2
76.	Dihydroaltersolanol C	C16H18O7	322.3	-0.2
77.	Fumiquinazoline N	C28H27N5O6	529.5	-0.2
78.	Serantrypinone	C21H16N4O4	388.3	-0.2
79.	Stemphylanthranol A	C48H40O21	952.8	-0.2
80.	Stemphylanthranol B	C48H40O21	952.8	-0.2
81.	Sphingofungin F	C21H39NO6	401.5	-0.2
82.	Gliovirin	C20H20N2O8S2	480.5	-0.2
83.	Porritoxinol	C15H24O6	300.3	-0.2
84.	Terreic acid	C7H6O4	154.1	-0.2
85.	T-2 tetraol	C15H22O6	298.3	-0.2
86.	T-2 triol	C20H30O7	382.4	-0.2
87.	Vermiculin	C20H24O8	392.4	-0.2
88.	3-Acetyldeoxynivalenol	C17H22O7	338.3	-0.1
89.	Altersolanol J	C16H20O6	308.3	-0.1
90.	Emestrin	C27H22N2O10S2	598.5	-0.1
91.	HC Toxin	C21H32N4O6	436.5	-0.1
92.	Myriocin	C21H39NO6	401.5	-0.1
93.	Pyrenocine B	C11H14O5	226.2	-0.1
94.	Cyclopiamide G	C15H16N2O3	272.3	0.0
95.	Brassicicene L	C20H30O5	350.4	0.0
96.	Deepoxydeoxynivalenol	C15H20O5	280.3	0.0
97.	Eremofortin C	C17H22O6	322.3	0.0
98.	Isoverrucarol	C15H22O4	266.3	0.0
99.	Pseurotin F2	C21H23NO8	431.4	0.0
100.	Slaframine	C10H18N2O2	198.2	0.0
101.	Verrucarol	C15H22O4	266.3	0.0
102.	Aflatoxicol M1	C17H14O7	330.2	0.1

103.	Aflatoxicol H1	C17H14O7	330.2	0.1
104.	Brassicicene E	C23H34O7	422.5	0.1
105.	Decarestrictine A	C10H14O4	198.2	0.1
106.	14-Norpseurotin A	C21H23NO8	417.4	0.1
107.	Sphyngofungin B	C20H39NO6	389.5	0.1
108.	Sporidesmin J	C17H18ClN3O6S2	459.9	0.1
109.	Aflatoxin M2	C17H14O7	330.2	0.2
110.	Bostrycin	C16H16O8	336.2	0.2
111.	Cyclochlorotine	C24H31Cl2N5O7	572.4	0.2
112.	Cyclopiamide H	C16H18N2O3	286.3	0.2
113.	Diacetoxyscirpenol	C19H26O7	366.4	0.2
114.	Gibberellic acid	C19H22O6	346.3	0.2
115.	5-Hydroxymaltol	C6H6O4	142.1	0.2
116.	Moniliformin	C4H2O3	98.0	0.2
117.	PR-Toxin	C17H20O6	320.3	0.2
118.	Radicinol	C12H14O5	238.2	0.2
119.	3-epi-Radicinol	C12H14O5	238.2	0.2
120.	Pyrenocine N	C10H12O4	196.2	0.2
121.	Pyrenocine O	C10H12O4	196.2	0.2
122.	Sporidesmin A	C18H20ClN3O6S2	473.9	0.2
123.	Brassicicene M	C21H32O5	364.4	0.3
124.	Fumiquinazoline E	C25H25N5O5	475.5	0.3
125.	7-Hydroxytrichodermol	C15H22O4	266.3	0.3
126.	Phomopsin A	C36H45ClN6O12	789.2	0.3
127.	Spinulosin	C8H8O5	184.1	0.3
128.	Aflatoxin GM	C17H14O8	346.2	0.4
129.	Dihydrogladiolic acid	C11H12O5	224.2	0.4
130.	Emestrin B	C27H22N2O10S3	630.6	0.4
131.	Fumiquinazoline O	C29H29N5O6	543.5	0.4
132.	Fumonisin C3	C33H57NO14	691.8	0.4
133.	HT-2 toxin	C22H32O8	424.4	0.4
134.	Koninginin I	C16H24O5	296.3	0.4
135.	Puberuline A	C22H29NO4	371.4	0.4
136.	Puberuline B	C22H29NO5	387.4	0.4
137.	Purpurogenone	C14H12O5	260.2	0.4
138.	Tetrahydroaltersolanol B	C16H20O6	308.3	0.4
139.	Tetrahydroaltersolanol C	C16H20O6	308.3	0.4

140.	Tetrahydroaltersolanol D	C16H20O6	308.3	0.4
141.	Tetrahydroaltersolanol E	C16H20O6	308.3	0.4
142.	Tyrosol	C8H10O2	138.1	0.4
143.	Aflatoxin M1	C17H12O7	328.2	0.5
144.	Aflatoxin Q1	C17H12O7	328.2	0.5
145.	Brassicicene D	C21H30O5	362.4	0.5
146.	Brassicicene G	C21H32O5	364.4	0.5
147.	Brassicicene N	C21H30O6	378.4	0.5
148.	Fumigatin	C8H8O4	168.1	0.5
149.	Pseurotin A	C22H25NO8	431.4	0.5
150.	Pseurotin A1	C22H25NO8	431.4	0.5
151.	Pyrenocine E	C12H16O5	240.2	0.5
152.	Sambucinol	C15H22O4	266.3	0.5
153.	Trichothecolone	C15H20O4	264.3	0.5
154.	Aflatoxin RB2	C17H20O6	320.3	0.6
155.	Brassicicene F	C21H32O5	364.4	0.6
156.	15-Decalonectrin	C17H24O5	308.4	0.6
157.	Eremofortin D	C17H24O6	324.3	0.6
158.	Fumiquinazoline A	C24H23N5O4	445.4	0.6
159.	Fumiquinazoline B	C24H23N5O4	445.4	0.6
160.	Koninginin J	C16H24O5	296.3	0.6
161.	Lysine ochratoxin A	C17H21ClN2O6	384.8	0.6
162.	Penicillic acid	C8H10O4	170.1	0.6
163.	Pyrenocin C	C11H14O4	210.2	0.6
164.	Pyrenocine I	C11H14O4	210.2	0.6
165.	Pyrenocine L	C14H20O6	284.3	0.6
166.	Trichoverrol A	C23H32O7	420.5	0.6
167.	Trichoverrol B	C23H32O7	420.5	0.6
168.	Viridic acid	C24H30N4O5	454.5	0.6
169.	Aflatoxin GM1	C17H12O8	344.2	0.7
170.	Altenuene	C15H16O6	292.2	0.7
171.	Azepinostatin	C28H26N2O10	550.5	0.7
172.	Decarestrictine C	C10H16O4	200.2	0.7
173.	Fumiquinazoline D	C24H21N5O4	443.4	0.7
174.	Fumiquinazoline M	C38H35N5O9	705.7	0.7
175.	Gladiolic acid	C11H10O5	222.1	0.7
176.	Glionitrin A	C13H11N3O5S2	353.3	0.7

177.	Isoaltenuene	C15H16O6	292.2	0.7
178.	Phomoeuphorbin B	C15H18O4	262.3	0.7
179.	Sporidesmin E	C18H20ClN3O6S3	506.0	0.7
180.	Puberuline C	C25H39NO6	449.5	0.7
181.	Pyrenocine D	C11H12O4	208.2	0.7
182.	Pyrenocine J	C11H14O4	210.2	0.7
183.	Aflatoxin B1 2,3-epoxide	C17H12O7	328.2	0.8
184.	Aflatoxin B2A	C17H14O7	330.2	0.8
185.	Alantrypinone	C21H16N4O3	372.3	0.8
186.	Altersolanol P	C15H14O5	274.2	0.8
187.	Aspochalasin U	C24H37NO5	419.5	0.8
188.	Chaetoglobin B	C38H48N2O12	724.8	0.8
189.	Cerulenin	C12H17NO3	223.2	0.8
190.	Cichorine	C10H11NO3	193.2	0.8
191.	Cyclopaldic acid	C11H10O6	238.1	0.8
192.	Dihydroaltersolanol B	C16H18O6	306.3	0.8
193.	Fumiquinazoline	C24H21N5O4	443.4	0.8
194.	Fumiquinazoline C	C24H21N5O4	443.4	0.8
195.	Fumiquinazoline P	C28H25N5O5	511.5	0.8
196.	Fumonisin B3	C34H59NO14	705.8	0.8
197.	Fumonisin C2	C33H57NO14	691.8	0.8
198.	Herbarin	C16H16O6	304.2	0.8
199.	Ochratoxin A cellobiose ester	C32H38ClNO16	728.0	0.8
200.	Talaroflavone	C18H16O8	360.3	0.8
201.	Aflatoxin RB1	C17H18O6	318.3	0.9
202.	Alterlosin II	C20H16O7	368.3	0.9
203.	Alternethanoxin C	C16H16O8	336.2	0.9
204.	Altersolanol B	C16H16O6	304.3	0.9
205.	Bostrycoidin	C15H11NO5	285.2	0.9
206.	Brevigellin	C31H41N5O7	595.6	0.9
207.	Carolic acid	C9H10O4	182.1	0.9
208.	Chrysogine	C10H10N2O2	190.2	0.9
209.	Brevione H	C27H30O7	466.5	0.9
210.	Dipodazine	C13H11N3O2	241.2	0.9
211.	Fumiquinazoline F-indoline-3'-ol	C21H19N4O3	375.4	0.9
212.	Scytalone	C10H10O4	194.1	0.9
213.	ACT-Toxin I	C26H39NO10	525.5	1.0

214.	Aflatoxin G1 9,10-epoxide	C17H12O8	344.3	1.0
215.	Aflatoxin G2A	C17H14O8	346.2	1.0
216.	Aflatoxin P2	C16H12O6	300.2	1.0
217.	Anacine	C18H22N4O3	342.3	1.0
218.	Brassicicene H	C21H34O4	350.4	1.0
219.	Chaetomugilin R	C16H21ClO5	328.7	1.0
220.	Diaportinol	C13H14O6	266.2	1.0
221.	Eremofortin A	C17H22O5	306.3	1.0
222.	Fusicoccin	C36H56O12	680.8	1.0
223.	Maculosin	C14H16N2O3	260.2	1.0
224.	Nigerapyrone E	C11H12O4	208.2	1.0
225.	Rugulosine	C30H22O10	542.4	1.0
226.	T-2 toxin	C24H34O9	466.5	1.0
227.	Aflatoxin B1 9-chloride	C17H13ClO7	364.7	1.1
228.	Aflatoxin M4	C17H12O7	328.2	1.1
229.	Alterlosin I	C20H14O7	366.3	1.1
230.	Austalide R	C25H32O9	476.5	1.1
231.	Citreoisocumarin	C14H14O6	278.2	1.1
232.	Cladospirone E	C20H16O6	352.3	1.1
233.	Cladospirone D	C20H14O7	366.3	1.1
234.	Cyclopenol	C17H14N2O4	310.3	1.1
235.	Cytromycetin	C14H10O7	290.2	1.1
236.	Dehydrocarolic acid	C9H8O4	180.1	1.1
237.	Globosuxanthone A	C15H12O7	304.2	1.1
238.	Koninginin D	C16H26O5	298.3	1.1
239.	Myrotoxin A	C27H32O9	500.5	1.1
240.	Nigrosporin A	C16H16O6	304.2	1.1
241.	Nigrosporin B	C16H16O6	304.2	1.1
242.	Pyrenocine A	C11H12O4	208.2	1.1
243.	Radicinin	C12H12O5	236.2	1.1
244.	3-Epi-Radicinin	C12H12O5	236.2	1.1
245.	Sporidesmin D	C20H26ClN3O6S2	504.0	1.1
246.	Sporidesmin G	C18H20ClN3O6S4	538.0	1.1
247.	Terreinol	C13H14O5	250.2	1.1
248.	Viridiol	C20H18O6	354.3	1.1
249.	Yanuthone I	C17H24O6	324.3	1.1
250.	Aflatoxin R0	C17H14O6	314.3	1.2

251.	Alternarienonic acid	C14H14O6	278.2	1.2
252.	Baccatin III	C31H38O11	586.6	1.2
253.	Brassicicene J	C21H32O6	380.4	1.2
254.	Calonectrin	C19H26O6	350.4	1.2
255.	4-Chromanol	C9H10O2	150.1	1.2
256.	Chromomycinone	C21H24O9	420.4	1.2
257.	Circumdatin I	C17H13N3O4	323.3	1.2
258.	Circumdatin K	C16H11N3O3	293.2	1.2
259.	Cladospirone C	C17H13N3O3	307.3	1.2
260.	Decaspirone H	C20H20O6	356.3	1.2
261.	Destruxin D2	C29H47N5O9	609.7	1.2
262.	Destruxin E	C29H47N5O8	593.7	1.2
263.	Dihydrofusarubin	C15H16O7	308.2	1.2
264.	Ethisolide	C9H10O4	182.1	1.2
265.	4-epi-Ethisolide	C9H10O4	182.1	1.2
266.	Fumonisin B2	C34H59NO14	705.8	1.2
267.	Maximiscin	C23H31NO8	449.5	1.2
268.	Parasiticol	C16H14O6	302.2	1.2
269.	Pestalamide C	C15H16N2O3	272.3	1.2
270.	Sambucoin	C15H22O3	250.3	1.2
271.	Satratoxin G	C29H36O10	544.5	1.2
272.	Spiroxin C	C20H10O7	362.2	1.2
273.	Sporidesmin B	C18H20ClN3O5S2	457.9	1.2
274.	*Terretonin*	C20H32O9	488.5	1.2
275.	Trichodermol	C15H22O3	250.3	1.2
276.	Wortmannin	C23H24O8	428.4	1.2
277.	Aflatoxin B2	C17H14O6	314.2	1.3
278.	Aflatoxin P1	C16H10O6	298.2	1.3
279.	Alternethanoxin D	C16H14O8	334.2	1.3
280.	Brassicicene B	C21H34O4	350.4	1.3
281.	Cyclopiamide D	C19H16N2O4	336.3	1.3
282.	Destruxin C2	C29H49N5O8	595.7	1.3
283.	Destruxin F	C29H49N5O8	595.7	1.3
284.	9,10-Dihydrolysergol	C16H20N2O	256.3	1.3
285.	Elymoclavine	C16H18N2O	254.3	1.3
286.	Emestrin H	C20H22N2O4S2	418.5	1.3
287.	Frequentin	C14H20O4	252.3	1.3

288.	Isosatratoxin	C29H34O10	542.6	1.3
289.	Luteoskyrin	C30H22O12	574.4	1.3
290.	Orsellide A	C15H18O8	326.3	1.3
291.	Orsellide B	C15H18O8	326.3	1.3
292.	Porriolide	C10H10O4	194.1	1.3
293.	Pyrenophorin	C16H20O6	308.3	1.3
294.	Satratoxin F	C29H34O10	542.5	1.3
295.	Satratoxin H	C29H36O9	528.5	1.3
296.	Stemphone F	C33H48O10	604.7	1.3
297.	Tryptoquivaline K	C25H20N4O5	456.4	1.3
298.	Verrucine A	C21H20N4O3	376.4	1.3
299.	Alterporriol A	C32H26O13	618.5	1.4
300.	Alterporriol B	C32H26O13	618.5	1.4
301.	Alterporriol C	C32H26O13	618.5	1.4
302.	Alterporriol G/H	C32H26O13	618.5	1.4
303.	Alterporriol N	C32H26O13	618.5	1.4
304.	Altertoxin I	C20H16O6	352.3	1.4
305.	Atranone H	C24H32O8	448.5	1.4
306.	Austin	C27H32O9	500.5	1.4
307.	Chaetomugilin Q	C22H29ClO6	424.9	1.4
308.	Chaetocyclinone A	C17H16O8	348.3	1.4
309.	Chaetocyclinone B	C16H14O7	318.2	1.4
310.	Cladospolide A	C12H20O4	228.2	1.4
311.	Cladospolide B	C12H20O4	228.2	1.4
312.	Cladospolide C	C12H20O4	228.2	1.4
313.	Daldinin F	C23H26O9	446.4	1.4
314.	4-Deoxybostrycin	C16H6O7	320.2	1.4
315.	Ergovaline	C29H35N5O5	533.6	1.4
316.	Fumitremorgen E	C22H18N4O5	418.4	1.4
317.	Fumitremorgen F	C22H18N4O4	402.4	1.4
318.	Fusarubin	C15H14O7	306.2	1.4
319.	Javanicin C	C22H34O7	410.5	1.4
320.	Norjavanicin	C14H12O6	276.2	1.4
321.	Palmarumycin C9	C20H10O7	362.2	1.4
322.	Penifulvin B	C15H20O5	280.3	1.4
323.	Preussomerin J	C20H10O7	362.3	1.4
324.	Pyrenocine M	C12H18O4	226.3	1.4

325.	Pyrenophorol	C16H24O6	312.4	1.4
326.	Roridin L	C29H38O9	530.6	1.4
327.	Rubratoxin A	C26H32O11	520.5	1.4
328.	Spiroxin E	C20H10Cl2O8	449.1	1.4
329.	Tryptoquialanone	C25H22N4O6	474.4	1.4
330.	Verrucarin L	C27H32O9	500.5	1.4
331.	Verrucin F	C21H18N4O3	374.4	1.4
332.	Acetyl-T-2 toxin	C26H36O10	508.5	1.5
333.	Aflatoxin G2	C17H14O7	330.3	1.5
334.	Amudol	C7H7ClO3	174.5	1.5
335.	Aspochalasin E	C24H37NO5	419.6	1.5
336.	Atranone F	C24H32O7	432.5	1.5
337.	Auranthine	C19H14N4O2	330.3	1.5
338.	Brassicicene A	C21H32O4	348.4	1.5
339.	Brassicicene C	C21H32O4	348.4	1.5
340.	Brevianamide F	C16H17N3O2	283.3	1.5
341.	Brevianamide M	C18H15N3O3	321.3	1.5
342.	Cladospirone G	C20H20O6	356.3	1.5
343.	Chaetochromone B	C14H14O5	262.2	1.5
344.	3-Chromanol	C9H10O2	150.1	1.5
345.	Cyclopenin	C17H14N2O3	294.3	1.5
346.	Cyclopiamide F	C15H12N2O2	252.2	1.5
347.	Desmethoxyviridiol	C19H16O5	324.3	1.5
348.	Fiscalin A	C26H27N5O4	473.5	1.5
349.	Palitantin	C14H22O4	254.3	1.5
350.	Phomapyrone B	C12H16O4	224.2	1.5
351.	Sclerotigenin	C16H11N3O2	277.2	1.5
352.	Stemphyperylenol	C20H16O6	352.3	1.5
353.	Trypacidin	C18H16O7	344.3	1.5
354.	Viridicatic acid	C12H16O6	256.2	1.5
355.	Abscisic acid	C15H20O4	264.3	1.6
356.	Aflatoxin B1	C17H12O6	312.3	1.6
357.	Aflatoxin D1	C16H14O5	286.3	1.6
358.	Alteichin	C20H14O6	350.3	1.6
359.	Alterperylenol	C20H14O6	350.3	1.6
360.	Aspergillide C	C14H20O4	252.3	1.6
361.	Austalide O	C26H34O9	490.5	1.6

362.	Brassicicene K	C21H30O4	346.4	1.6
363.	Cyclopiamide C	C19H18N2O4	338.3	1.6
364.	Circumdatin C	C17H13N3O3	307.3	1.6
365.	Circumdatin G	C17H13N3O3	307.3	1.6
366.	Circumdatin L	C17H13N3O3	307.3	1.6
367.	Crotocin	C19H24O5	332.3	1.6
368.	Dechlorogriseofulvine	C17H18O6	318.3	1.6
369.	7-Dehydrobrefeldin A	C16H22O4	278.3	1.6
370.	Deoxyradicinin	C12H12O4	220.2	1.6
371.	Destruxin A1	C30H49N5O7	591.7	1.6
372.	Destruxin C	C30H51N5O8	609.7	1.6
373.	Dethiosecoemestrin	C27H20N2O10	532.4	1.6
374.	epi-Dechlorogriseofulvin	C17H18O6	318.3	1.6
375.	Ergin	C16H17N3O	267.3	1.6
376.	Fusarochromanone	C15H20N2O4	292.3	1.6
377.	Fumitremorgen G	C23H20N4O5	432.4	1.6
378.	Ganomastenol C	C15H26O3	254.3	1.6
379.	Glionitrin B	C15H17N3O5S2	383.4	1.6
380.	Gregatin E	C14H18O5	266.2	1.6
381.	Koninginin F	C16H26O5	298.3	1.6
382.	Paragerquamide D	C28H33N3O5	491.5	1.6
383.	Paragerquamide I	C28H31N3O5	489.5	1.6
384.	Pestalotin	C11H18O4	214.2	1.6
385.	Phomapyrone D	C12H14O4	222.2	1.6
386.	Pyrophen	C16H17NO4	287.3	1.6
387.	Radicinin monoacetate	C14H14O6	278.2	1.6
388.	Terrestrol C	C14H14O5	262.2	1.6
389.	Walleminone	C15H24O3	252.3	1.6
390.	Arisugacin J	C27H32O8	484.5	1.7
391.	Aspergillic acid	C12H20N2O2	224.3	1.7
392.	Aspergillide A	C14H22O4	254.3	1.7
393.	Aspergillide B	C14H22O4	254.3	1.7
394.	Atranone G	C25H34O8	462.5	1.7
395.	Bis-Dechlorogeodin	C17H14O7	330.2	1.7
396.	Chaetoviridin D	C23H29ClO8	468.9	1.7
397.	Chaetoviridin I	C23H27ClO8	466.9	1.7
398.	Cladospolide D	C12H18O4	226.2	1.7

399.	Cyclopiamide	C16H14N2O2	266.3	1.7
400.	Daldinin A	C15H18O5	278.3	1.7
401.	Dehydroaltenusin	C15H12O6	288.2	1.7
402.	Desmethyldestruxin A	C27H43N5O7	549.6	1.7
403.	Dihydrotentoxin	C22H32N4O4	416.5	1.7
404.	Fiscalin C	C27H29N5O4	487.5	1.7
405.	Eremofortin B	C15H20O3	248.3	1.7
406.	Fumagillol	C16H26O4	282.3	1.7
407.	Fusarin X	C22H29NO5	387.4	1.7
408.	Fusarinolic acid	C10H12NO3	194.2	1.7
409.	Hydroxyproline ochratoxin A	C16H16ClNO7	369.7	1.7
410.	Isomarticin	C18H16O9	376.3	1.7
411.	Koninginin G	C16H30O5	302.4	1.7
412.	Malformin A2	C22H37N5O5S2	515.6	1.7
413.	Marticin	C18H16O9	376.3	1.7
414.	Orsellide D	C14H14O7	294.2	1.7
415.	Paragerquamide A	C28H35N3O5	493.6	1.7
416.	Patulodin	C23H26O8	430.4	1.7
417.	Preussomerin E	C20H12O7	364.3	1.7
418.	Quinolactacin C	C16H18N2O3	286.3	1.7
419.	Roquefortine L	C22H21N5O3	403.4	1.7
420.	Rubroskyrin	C30H22O12	574.4	1.7
421.	Vertinolide	C14H18O4	250.2	1.7
422.	Yanuthone J	C28H42O9	522.6	1.7
423.	Aflatoxin G1	C17H12O7	328.2	1.8
424.	Alternarian acid	C15H12O8	320.2	1.8
425.	Atranone I	C24H32O7	432.5	1.8
426.	Austalide F	C26H34O9	490.5	1.8
427.	Circumdatin D	C21H19N3O5	393.3	1.8
428.	Circumdatin E	C20H17N3O4	363.3	1.8
429.	Cladosporone A	C20H16O6	352.3	1.8
430.	Cyclopiamide B	C20H20N2O4	352.3	1.8
431.	Fumiquinazoline L	C26H25N5O4	471.5	1.8
432.	Ergometrine	C19H23N3O2	325.4	1.8
433.	Ergometrinine	C19H23N3O2	325.4	1.8
434.	Ergosine	C30H37N5O5	547.6	1.8
435.	Ergosinine	C30H37N5O5	547.6	1.8

436.	Gregatin D	C16H22O5	294.3	1.8
437.	Lumpidine	C26H28N4O4	460.5	1.8
438.	Neoaspergillic acid	C12H20N2O2	224.3	1.8
439.	Questinol	C12H12O6	300.3	1.8
440.	Penicillin G	C16H18N2O4S	334.3	1.8
441.	Roridin J	C29H36O9	528.5	1.8
442.	Preussomerin H	C20H12O7	364.3	1.8
443.	Trichoverrin A	C29H40O9	532.6	1.8
444.	Trichoverrin B	C29H40O9	532.6	1.8
445.	Trichodermin	C17H24O4	292.3	1.8
446.	Tryptophol	C10H11NO	161.2	1.8
447.	Verruculogen	C27H33N3O7	511.5	1.8
448.	Yanuthone G	C28H42O9	522.6	1.8
449.	Asperthecin	C15H10O8	318.2	1.9
450.	Atranone J	C23H32O6	404.5	1.9
451.	Brevianamide A	C21H23N3O3	365.4	1.9
452.	Brevianamide B	C21H23N3O3	365.4	1.9
453.	Brevianamide U	C21H23N3O4	381.4	1.9
454.	Brevione E	C28H34O7	482.5	1.9
455.	Chaetoglobin A	C34H40N2O10	636.6	1.9
456.	Canescin A	C15H14O7	306.2	1.9
457.	Canescin B	C15H14O7	306.2	1.9
458.	Chaetomine	C31H30N6O6S4	710.8	1.9
459.	2-Chromanol	C9H10O2	150.1	1.9
460.	Circumdatin F	C17H13N3O2	291.3	1.9
461.	Decaspirone A	C20H16O5	336.3	1.9
462.	Decaspirone F	C20H18O5	338.3	1.9
463.	Decaspirone G	C20H18O5	338.3	1.9
464.	Desmethyldestruxin B2	C27H45N5O7	551.6	1.9
465.	Emodic acid	C15H8O7	300.2	1.9
466.	Fumiquinazoline I	C27H29N5O4	487.5	1.9
467.	15alpha-Hydroxyculmorin	C15H26O3	254.4	1.9
468.	11-Hydroxycurvularin	C16H20O6	308.3	1.9
469.	Javanicin B	C21H28O6	376.4	1.9
470.	Nectriafurone	C15H12O7	304.2	1.9
471.	Nigerapyrone C	C13H14O4	234.2	1.9
472.	Nigragillin	C13H22N2O	222.3	1.9

473.	Orsellide C	C15H18O7	310.3	1.9
474.	Palmarumycin P2	C20H16O5	336.3	1.9
475.	Paragerquamide H	C28H33N3O5	491.5	1.9
476.	Pestalamide A	C18H17NO6	343.3	1.9
477.	Pestalamide B	C18H18N2O5	342.3	1.9
478.	Puberuline F	C16H15NO6	317.2	1.9
479.	Roridin M	C29H36O9	528.5	1.9
480.	Rugulovasine A	C16H16N2O2	268.3	1.9
481.	Rugulovasine B	C16H16N2O2	268.3	1.9
482.	Secoemestrin D	C27H26N2O8	506.5	1.9
483.	Solanapyrone F	C17H21NO4	303.3	1.9
484.	Sorrentanone	C14H14O4	246.2	1.9
485.	Sporidesmin H	C18H20ClN3O4S2	441.9	1.9
486.	Terrestric acid	C11H14O4	210.2	1.9
487.	Trichothecin	C19H24O5	332.3	1.9
488.	Tricycloalternarene E	C21H32O5	364.4	1.9
489.	ACRL Toxin II A	C17H24O5	308.3	2.0
490.	Altechromone B	C14H14O4	246.2	2.0
491.	Alternethanoxin A	C16H14O6	302.2	2.0
492.	Alternethanoxin B	C16H12O6	302.2	2.0
493.	Alternethanoxin E	C16H12O6	302.2	2.0
494.	Ascotoxin (Brefeldin A)	C16H24O4	280.3	2.0
495.	Aspochalasin H	C24H35NO5	417.5	2.0
496.	Atranone K	C24H32O8	448.5	2.0
497.	Austocystin F	C17H10O7	326.2	2.0
498.	Chaetocin	C30H28N6O6S4	696.8	2.0
499.	Chaetomugilin A	C23H27ClO7	450.9	2.0
500.	Chaetomugilin M	C23H27ClO7	450.9	2.0
501.	Canadensolide	C11H14O4	210.2	2.0
502.	Citrinin	C13H14O5	250.2	2.0
503.	Cyclopiamide E	C20H17N3O2	331.3	2.0
504.	Epoxyagroclavine	C16H18N2O	254.3	2.0
505.	Ergotamine	C33H35N5O5	581.6	2.0
506.	Ergotaminine	C33H35N5O5	581.6	2.0
507.	Fumonisin A1	C36H61NO16	763.8	2.0
508.	15-Hydroxyculmorin	C15H26O3	254.4	2.0
509.	Infectopyrone A	C14H16O6	280.2	2.0

510.	Isotentoxin	C22H30N4O4	414.5	2.0
511.	Javanicin	C15H14O6	290.2	2.0
512.	Mitorubrinol	C21H18O8	398.3	2.0
513.	Nidurufin	C20H16O8	384.3	2.0
514.	Ochratoxin beta	C11H10O5	222.1	2.0
515.	Roridin D	C29H38O9	530.6	2.0
516.	Rubratoxin B	C26H30O11	518.5	2.0
517.	Sphyngofungin A	C21H41N3O6	431.5	2.0
518.	Spiroxin A	C20H9ClO8	412.7	2.0
519.	Tagetenolone	C16H22O5	294.3	2.0
520.	Tentoxin	C22H30N4O4	414.5	2.0
521.	Terrestrol E	C14H14O5	262.2	2.0
522.	Walleminol	C15H24O2	236.3	2.0
523.	Zinniol	C15H22O4	266.3	2.0
524.	ACT-Toxin II	C26H39NO9	509.5	2.1
525.	AF-toxin I	C17H24O6	324.3	2.1
526.	Ascochitine	C15H16O5	276.2	2.1
527.	Aspertoxin	C19H14O7	354.3	2.1
528.	Aspochalasin K	C25H39NO5	433.5	2.1
529.	Asteltoxin	C23H30O7	418.4	2.1
530.	Brevianamide E	C21H25N3O3	367.4	2.1
531.	Diaporthin	C13H14O5	250.2	2.1
532.	Dihydroergosine	C30H39N5O5	549.6	2.1
533.	Fumiquinazoline H	C27H27N5O4	485.5	2.1
534.	Fumonisin C4	C33H57NO13	675.8	2.1
535.	6-Hydroxymellein	C10H10O4	194.1	2.1
536.	Malformin A	C23H39N5O5S2	529.7	2.1
537.	Malformin A1 E	C23H39N5O5S2	529.7	2.1
538.	Palmarumycin P4	C20H16O5	336.3	2.1
539.	Penicillin	C16H18N2O4S	334.3	2.1
540.	Porritoxin	C17H23NO4	305.3	2.1
541.	Puberuline E	C15H13NO6	303.2	2.1
542.	Quinolactacin B	C15H16N2O2	256.3	2.1
543.	Serine ochratoxin A	C14H14ClNO7	343.7	2.1
544.	Stemphyltoxin III	C20H12O6	348.3	2.1
545.	Tenuazonic acid	C10H15NO3	197.2	2.1
546.	Terrestrol H	C15H16O5	276.2	2.1

547.	Tricycloalternarene 2a	C21H30O4	346.4	2.1
548.	ACRL Toxin IIIB	C20H30O6	366.4	2.2
549.	AF-toxin II	C17H24O6	324.3	2.2
550.	AF-toxin IIc	C17H24O6	324.3	2.2
551.	Alternaric acid	C21H30O8	410.4	2.2
552.	Aspochalasin O	C27H39NO5	457.6	2.2
553.	Aspterric acid	C15H22O4	266.3	2.2
554.	Austalide M	C27H36O9	504.5	2.2
555.	Austalide N	C28H36O10	532.5	2.2
556.	Avenacein Y	C15H10O8	318.2	2.2
557.	Brevianamide N	C18H13N3O3	319.3	2.2
558.	Chanoclavine-I	C16H20N2O	256.3	2.2
559.	Chanoclavine-II	C16H20N2O	256.3	2.2
560.	Circumdatin H	C20H17N3O3	347.3	2.2
561.	Circumdatin J	C21H19N3O4	377.4	2.2
562.	Clavatol	C10H12O3	180.2	2.2
563.	Cytochalasin C	C30H37NO6	507.6	2.2
564.	Decaspirone E	C22H18O6	378.3	2.2
565.	Fonsecin	C15H14O6	290.2	2.2
566.	Fumigaclavine B	C16H20N2O	256.3	2.2
567.	Gregatin C	C16H22O5	294.3	2.2
568.	Griseofulvine	C17H17ClO6	352.7	2.2
569.	Isochanoclavine	C17H22N2O	270.3	2.2
570.	Isofumigaclavine B	C16H20N2O	256.3	2.2
571.	Javanicin A	C21H30O6	378.4	2.2
572.	Lambertellin	C14H8O5	256.2	2.2
573.	Lysergol	C16H18N2O	254.3	2.2
574.	Mitorubrinic acid	C21H16O9	412.4	2.2
575.	Nigerapyrone C	C13H14O4	234.2	2.2
576.	Ochratoxin quinone	C20H17NO7	383.4	2.2
577.	Orsellide E	C14H14O6	278.2	2.2
578.	Palmarumycin CE1	C20H18O5	338.3	2.2
579.	Palmarumycin CE2	C20H20O5	340.3	2.2
580.	Palmarumycin CE3	C20H18O5	338.3	2.2
581.	Palmarumycin CR1	C20H20O5	340.3	2.2
582.	Phomapyrone G	C14H18O4	250.3	2.2
583.	Puberuline D	C18H19NO7	361.3	2.2

584.	Roridin A	C29H40O9	532.6	2.2
585.	Koninginin E	C16H26O4	282.3	2.2
586.	Aspulvinone G	C17H12O6	312.2	2.3
587.	Aurantiamine	C16H22N4O2	302.3	2.3
588.	Aureonitol	C13H18O2	206.2	2.3
589.	Austocystin I	C18H12O7	340.2	2.3
590.	Barceloneic acid A	C16H16O7	320.2	2.3
591.	Chaetoglobosine O	C32H38N2O5	530.7	2.3
592.	Chromomycin A3	C57H82O26	1183.2	2.3
593.	Destruxin A	C29H47N5O7	577.7	2.3
594.	Diplodiatoxin	C18H28O4	308.4	2.3
595.	Ergoline	C14H16N2	212.2	2.3
596.	Fumitremorgen N	C26H24N4O5	472.5	2.3
597.	Fusarin Z	C22H25NO7	415.4	2.3
598.	5'-Hydroxyasperentin	C16H20O6	308.3	2.3
599.	Insulicolide A	C22H25NO8	431.4	2.3
600.	Italicic acid	C15H16O6	292.2	2.3
601.	Metisergid	C21H27N3O2	353.4	2.3
602.	Monacolin J	C19H28O4	320.4	2.3
603.	Penochalasin H	C32H38N2O5	530.6	2.3
604.	Phomapyrone E	C14H18O4	250.2	2.3
605.	Phomopsichalasin B	C28H35NO4	449.5	2.3
606.	Terrestrol B	C14H13ClO5	296.7	2.3
607.	Topopyrone D	C18H10O7	338.2	2.3
608.	Versicolorin B	C18H12O7	340.2	2.3
609.	Verruculotoxin	C15H20N2O	244.3	2.3
610.	Viridamine	C16H22N4O2	302.3	2.3
611.	Viridicatumtoxin	C30H31NO10	565.5	2.3
612.	Viriditoxin	C30H31NO10	565.5	2.3
613.	Visoltricin	C13H18N2O2	234.2	2.3
614.	Aflatoxin B1 2,3-dichloride	C17H12Cl2O6	383.1	2.4
615.	Alterlactone	C15H12O6	288.2	2.4
616.	Anhydrofusarubin	C15H12O6	288.2	2.4
617.	Austalide D	C28H36O10	532.5	2.4
618.	Austalide E	C28H36O10	532.5	2.4
619.	Barnol	C10H14O3	182.2	2.4
620.	Chaetomugilin G	C23H25ClO6	416.8	2.4

621.	Chaetomugilin P	C22H27ClO5	406.9	2.4
622.	Chaetoviridin H	C22H27ClO4	390.9	2.4
623.	Cyclopeptin	C17H16N2O2	280.4	2.4
624.	Cytoglobosin C	C32H38N2O5	530.6	2.4
625.	Decaspirone B	C22H20O6	380.3	2.4
626.	Dihydroergotamine	C33H37N5O5	583.6	2.4
627.	Duclauxin	C29H22O11	546.4	2.4
628.	11-Epi-Chaetomugilin I	C22H27ClO5	406.9	2.4
629.	beta-9,10-Epoxy roridin H	C29H36O9	528.5	2.4
630.	Ergocornin	C31H39N5O5	561.6	2.4
631.	Ergocorninin	C31H39N5O5	561.6	2.4
632.	Falconensin I	C22H24O7	400.4	2.4
633.	Fonsecin B	C15H14O6	290.2	2.4
634.	Fumonisin B4	C34H59NO13	689.8	2.4
635.	Fusarin D	C23H29NO6	431.4	2.4
636.	Malformin C	C23H39N5O5S2	529.7	2.4
637.	Neoxaline	C23H25N5O4	435.4	2.4
638.	Ochratoxin A glucose ester	C26H28ClNO11	565.9	2.4
639.	Paragerquamide B	C27H33N3O4	463.5	2.4
640.	Patulolide A	C12H18O3	210.2	2.4
641.	Patulolide B	C12H18O3	210.2	2.4
642.	Phomopsichalasin A	C28H37NO4	451.6	2.4
643.	Roridin E	C29H38O8	514.6	2.4
644.	Roridin P	C29H38O8	514.6	2.4
645.	Sclerotide A	C37H39N7O8	709.7	2.4
646.	Sclerotide B	C37H39N7O8	709.7	2.4
647.	Tagetolone	C16H24O4	280.3	2.4
648.	Tricycloalternarene 1a	C21H32O4	348.4	2.4
649.	Verrucarin A	C27H34O9	502.5	2.4
650.	Viridicatin	C15H11NO2	237.2	2.4
651.	Xantocillin Y2	C18H12N2O4	320.3	2.4
652.	AF-toxin IIa methyl ester	C18H26O4	338.4	2.5
653.	*AK-Toxin II*	C22H25NO6	399.4	2.5
654.	Altenuisol	C14H10O6	274.2	2.5
655.	Altertenuol	C14H10O6	274.2	2.5
656.	Alterporriol P	C32H26O12	602.5	2.5
657.	Aspochalasin C	C24H35NO4	401.5	2.5

658.	Aspochalasin D	C24H35NO4	401.5	2.5
659.	Aspochalasin I	C24H35NO4	401.5	2.5
660.	Aspochalasin M	C24H35NO4	401.5	2.5
661.	Aspochalasin P	C24H35NO4	401.5	2.5
662.	Austamide	C21H21N3O3	363.4	2.5
663.	Austocystin D	C22H20O8	412.3	2.5
664.	Brevianamide C	C21H23N3O3	365.4	2.5
665.	Brevianamide L	C22H23N3O4	393.4	2.5
666.	Brevioxime	C15H22N2O3	278.3	2.5
667.	Cercophorin A	C20H16O9	400.3	2.5
668.	Chaetocyclinone C	C32H24O14	632.5	2.5
669.	Chaetomugilin B	C24H29ClO7	464.9	2.5
670.	Circumdatin A	C21H19N3O5-	393.3	2.5
671.	Circumdatin B	C20H17N3O4-	393.3	2.5
672.	Decaspirone D	C22H18O6	378.3	2.5
673.	Decaspirone E	C22H18O6	378.3	2.5
674.	Dehydrocyclopeptin	C17H14N2O2	278.3	2.5
675.	Emestrin J	C27H24N2O7S2	552.6	2.5
676.	Falconensin J	C22H26O7	402.4	2.5
677.	Fumiquinazoline F	C21H18N4O2	358.4	2.5
678.	Emodin triacetate	C21H16O8	396.3	2.5
679.	ent-Fumiquinazoline G	C21H18N4O2	358.4	2.5
680.	Mitorubrinol acetate	C23H20O9	440.4	2.5
681.	Monacolin M	C23H34O6	406.5	2.5
682.	Nectriapyrone	C11H14O3	194.2	2.5
683.	Palmarumycin P1	C21H18O6	366.3	2.5
684.	Patulolide C	C12H20O3	212.2	2.5
685.	Quinolactacin A1	C16H18N2O2	270.3	2.5
686.	Quinolactacin A2	C16H18N2O2	270.3	2.5
687.	Quinolactacin A	C16H18N2O2	270.3	2.5
688.	Taxol	C47H51NO14	853.9	2.5
689.	Territrem C	C28H32O9	512.5	2.5
690.	Verrucarin J	C27H32O9	500.5	2.5
691.	Agroclavine	C16H18N2	238.3	2.6
692.	Arisugacin H	C29H36O9	528.5	2.6
693.	Arugosin F	C15H12O5	272.2	2.6
694.	Aspulvinone E	C17H12O5	296.2	2.6

695.	Bicycloalternarene 3	C21H32O4	348.5	2.6
696.	Chaetoglobosine B	C32H36N2O5	528.6	2.6
697.	Chaetoglobosine G	C32H36N2O5	528.6	2.6
698.	Citreoviridin A	C23H30O6	402.4	2.6
699.	Corynesidone C	C15H12O6	288.2	2.6
700.	Cytoglobosin B	C23H34O6	530.6	2.6
701.	Desacetylpebrolide	C22H28O6	388.4	2.6
702.	Destruxin A4	C30H49N5O7	591.7	2.6
703.	Expansolide A	C19H26O5	334.4	2.6
704.	Expansolide B	C19H26O5	334.4	2.6
705.	Fusaric acid	C4H4O4	116.0	2.6
706.	omega-Hydroxypachybasin	C15H10O4	254.2	2.6
707.	Nortryptoquivaline	C28H28N4O7	532.5	2.6
708.	Roridin Q	C35H46O11	642.7	2.6
709.	Spiroxin B	C20H8Cl2O8	447.1	2.6
710.	Terrestrol D	C14H13ClO5	296.7	2.6
711.	Versicolorin A	C18H10O7	338.2	2.6
712.	Zinnimidine	C15H19NO3	261.3	2.6
713.	AM Toxin III	C22H29N3O6	431.4	2.7
714.	Arisugacin K	C27H32O7	468.5	2.7
715.	Barceloneic acid B	C16H14O8	334.2	2.7
716.	Brevianamide O	C22H23N3O4	393.4	2.7
717.	Chaetoglobosine Q	C32H38N2O6	546.6	2.7
718.	Chaetoglobosine R	C32H38N2O6	546.6	2.7
719.	Cytochalasin D	C30H37NO6	507.6	2.7
720.	Dermoglaucin	C16H12O6	300.2	2.7
721.	Emodin	C15H10O5	270.2	2.7
722.	Ergocryptine	C32H40BrN5O5	654.6	2.7
723.	Ergocryptinnine	C32H41N5O5	575.7	2.7
724.	Falconensin F	C23H26O7	414.4	2.7
725.	Fumitremorgen B	C27H33N3O5	479.5	2.7
726.	Fusarin PM	C22H27NO7	417.4	2.7
727.	Globosuxanthone D	C14H8O5	256.2	2.7
728.	Griseophenone D	C15H14O6	290.2	2.7
729.	20-Hydroxyfusarin	C22H29NO6	403.4	2.7
730.	Infectopyrone	C14H16O5	264.2	2.7
731.	(4Z)-Infectopyrone	C14H16O5	264.2	2.7

732.	Koninginin A	C16H28O4	284.3	2.7
733.	Marcfortine B	C27H33N3O4	463.5	2.7
734.	3-Methylviridicatin	C16H13NO2	251.2	2.7
735.	3-Methoxyviridicatin	C16H13NO2	251.2	2.7
736.	Nalgiovensin	C18H16O6	328.3	2.7
737.	Paragerquamide C	C29H35N3O4	489.6	2.7
738.	Pyripyropene C	C32H39NO10	597.7	2.7
739.	3-O-Methylfunicone	C20H20O8	388.3	2.7
740.	Phomoxanthone A	C38H38O16	750.7	2.7
741.	Phomoxanthone B	C38H38O16	750.7	2.7
742.	Roridin R	C29H38O9	530.6	2.7
743.	Sphingofungin D	C22H41NO7	431.5	2.7
744.	Terrestrol F	C14H13ClO5	296.7	2.7
745.	Terrestrol G	C14H13ClO5	296.7	2.7
746.	Territrem A	C28H30O9	510.5	2.7
747.	Tricycloalternarene 2b	C21H30O4	346.4	2.7
748.	Yanuthone H	C22H32O5	376.4	2.7
749.	AK-toxin I	C23H27NO6	413.4	2.8
750.	Aspochalasin B	C24H33NO4	399.5	2.8
751.	Aspochalasin N	C27H39NO4	441.6	2.8
752.	Arisugacin A	C28H32O8	496.5	2.8
753.	Arisugacin B	C27H30O7	466.5	2.8
754.	Austalide B	C26H34O8	474.5	2.8
755.	Austocystin E	C23H22O8	426.4	2.8
756.	Chaetomugilin C	C23H25ClO6	432.8	2.8
757.	Chaetoxanthone A	C20H18O7	370.3	2.8
758.	Cyclopiamine A	C26H33N3O5	467.5	2.8
759.	Cyclopiamine B	C26H33N3O5	467.5	2.8
760.	Cytochalasin J	C28H37NO4	451.6	2.8
761.	Destruxin B	C30H51N5O7	593.7	2.8
762.	Fumigaclavine A	C18H22N2O2	298.3	2.8
763.	Globosuxanthone C	C14H10O5	258.2	2.8
764.	Gregatin B	C14H18O4	250.2	2.8
765.	Isofumigaclavine A	C18H23N2O2$^+$	299.3	2.8
766.	Marcfortine A	C28H35N3O4	477.6	2.8
767.	Norlichexanthone	C14H10O5	258.2	2.8
768.	Penifulvin A	C15H20O4	264.3	2.8

769.	Porric acid B	C15H12O8	288.2	2.8
770.	Porric acid C	C14H10O5	258.2	2.8
771.	Pyripyropene D	C32H39NO10	597.7	2.8
772.	Ravenelin	C14H10O5	258.2	2.8
773.	Roquefortine A	C18H22N2O2	298.3	2.8
774.	Roquefortine I	C23H25N5O3	419.4	2.8
775.	Rugulosuvine	C20H19N3O2	333.4	2.8
776.	Territrem B	C29H34O9	526.5	2.8
777.	Tryptoquivaline	C29H30N4O7	546.5	2.8
778.	Vincristine	C46H56N4O10	824.9	2.8
779.	Xantocillin Y1	C18H12N2O3	304.3	2.8
780.	Altenusin	C15H14O6	290.2	2.9
781.	Alternariol	C14H10O5	258.2	2.9
782.	Austalide C	C30H38O11	574.6	2.9
783.	Austocystin G	C18H11ClO7	374.7	2.9
784.	Brevianamide Q	C21H23N3O3	365.4	2.9
785.	Catenarin	C15H10O6	286.2	2.9
786.	Chaetoglobosine E	C32H38N2O6	530.6	2.9
787.	Chaetomugilin N	C23H25ClO6	432.8	2.9
788.	Cynodontin	C15H10O6	286.2	2.9
789.	Desertorin A	C22H18O8	410.3	2.9
790.	Fusarielin B	C25H40O5	420.6	2.9
791.	Monocerin	C16H20O6	308.3	2.9
792.	Orlandin	C22H18O8	410.3	2.9
793.	Paragerquamide E	C28H35N3O4	477.6	2.9
794.	Phomopsichalasin C	C30H39NO6	509.6	2.9
795.	Purpurin	C14H8O5	256.2	2.9
796.	Pyripyropene A	C31H37NO10	583.6	2.9
797.	Pyripyropene B	C32H39NO10	597.6	2.9
798.	Roquefortine D	C22H25N5O2	391.4	2.9
799.	Roquefortine G	C29H35N5O4	517.6	2.9
800.	Roridin H	C29H36O8	512.6	2.9
801.	Terpestacin	C25H38O4	402.6	2.9
802.	Topopyrone C	C18H10O7	338.3	2.9
803.	Verrucosidin	C24H32O6	416.5	2.9
804.	AM Toxin I	C23H31N3O6	445.5	3.0
805.	Asperentin 8-O-methylether	C17H22O5	306.4	3.0

806.	Asterric acid	C17H12O8	348.3	3.0
807.	Averufin	C20H16O7	368.3	3.0
808.	Averufanin	C20H18O7	370.4	3.0
809.	Bicycloalternarene 9	C21H32O4	348.5	3.0
810.	Brevione D	C27H32O5	436.6	3.0
811.	Brevione F	C27H32O5	436.6	3.0
812.	Brevione G	C27H32O5	436.6	3.0
813.	Citreoviridin D	C24H32O6	416.5	3.0
814.	Cladospirone B	C20H14O6	350.3	3.0
815.	Corynesidone A	C15H12O5	272.3	3.0
816.	Corynesidone D	C16H12O7	316.3	3.0
817.	Culmoron	C15H24O2	236.4	3.0
818.	Dehydrocurvularin	C16H18O5	290.3	3.0
819.	Destruxin A5	C31H51N5O7	605.7	3.0
820.	Emodin 3-methyl ether (Physcione)	C16H12O5	284.3	3.0
821.	Ergine	C16H17N3O	267.3	3.0
822.	Ergoctistin	C35H39N5O5	609.7	3.0
823.	Ergocristinin	C35H39N5O5	609.7	3.0
824.	Falconensin K	C22H23ClO7	434.8	3.0
825.	Fumitremorgin C	C22H25N3O3	379.5	3.0
826.	Fumonisin FP1	C39H62NO16	800.9	3.0
827.	Fusarin C	C23H29NO7	431.5	3.0
828.	epi-Fusarin C	C23H29NO7	431.5	3.0
829.	Geodin	C17H12Cl2O7	399.2	3.0
830.	Macrosporin	C16H12O5	284.3	3.0
831.	Marcfortine C	C27H33N3O3	447.6	3.0
832.	Meleagrin	C23H23N5O4	433.5	3.0
833.	Meleagrin E	C32H39N5O6	589.7	3.0
834.	Lysergide (LSD)	C20H25N3O	323.4	3.0
835.	Questin (Emodin 3-methyl ether)	C16H12O5	284.3	3.0
836.	Roquefortine C	C22H23N5O2	389.5	3.0
837.	Rubrofusarin	C15H12O5	272.3	3.0
838.	Stachybotrin A	C23H31NO5	401.5	3.0
839.	Terpendole I	C27H35NO5	453.6	3.0
840.	Topopyrone B	C18H9ClO7	372.7	3.0
841.	Tricycloalternarene 1b	C21H32O4	348.5	3.0

842.	Verrucarin K	C27H34O8	486.6	3.0
843.	Zearalenone-4-sulfate	C18H22O8S	398.4	3.0
844.	Yanuthone E	C28H40O8	504.6	3.0
845.	Aspochalasin F	C23H31NO4	385.5	3.1
846.	Aspochalasin G	C23H31NO3	369.5	3.1
847.	Aspochalasin Q	C24H35NO3	385.6	3.1
848.	Austalide J	C25H32O7	444.5	3.1
849.	AM Toxin II	C22H29N3O5	415.5	3.1
850.	Bisvertinol	C28H34O8	498.6	3.1
851.	Byssochlamic acid	C18H20O6	332.4	3.1
852.	Curvularin	C16H20O5	292.3	3.1
853.	Cytoglobosin A	C32H36N2O5	528.7	3.1
854.	Cytoglobosin E	C32H36N2O5	528.7	3.1
855.	Dichlorodiaportin	C12H12Cl2O5	319.1	3.1
856.	Dihydroxysterigmatocystin	C18H14O6	326.3	3.1
857.	Falconensin L	C22H25ClO7	436.9	3.1
858.	Griseophenone C	C16H16O6	304.3	3.1
859.	Janthitrem E	C37H49NO6	603.8	3.1
860.	Janthitrem F	C39H51NO7	645.8	3.1
861.	O-Methylsterigmatocystin	C19H14O6	338.3	3.1
862.	Pachybasic acid	C15H8O5	268.2	3.1
863.	Patulolide C acetate	C14H22O4	254.3	3.1
864.	alpha-Paxitriol	C27H35NO4	437.6	3.1
865.	beta-Paxitriol	C27H35NO4	437.6	3.1
866.	Petrosifungin B	C33H47N5O7	625.8	3.1
867.	Phomapyrone F	C14H16O3	232.3	3.1
868.	Phomarin	C15H10O4	254.2	3.1
869.	Porric acid A	C16H14O6	302.3	3.1
870.	Roquefortine H	C28H33N5O3	487.6	3.1
871.	Secalonic acid A	C32H30O14	638.6	3.1
872.	Secalonic acid B	C32H30O14	638.6	3.1
873.	Secalonic acid E	C32H30O14	638.6	3.1
874.	Secalonic acid D	C32H30O14	638.6	3.1
875.	Secalonic acid G	C32H30O14	638.6	3.1
876.	Trachyspic acid	C20H28O9	412.4	3.1
877.	Xanalteric acid II	C20H12O7	364.3	3.1
878.	Xantocillin	C18H12N2O2	288.3	3.1

879.	3-O-Acetoxypaxilline	$C_{29}H_{37}NO_5$	479.6	3.2
880.	19-O-Acetylchaetoglobosin B	$C_{34}H_{38}N_2O_6$	570.7	3.2
881.	Alternariol methyl ester	$C_{15}H_{12}O_5$	272.3	3.2
882.	Altertoxin VI	$C_{20}H_{12}O_5$	332.3	3.2
883.	Aurantioclavine	$C_{15}H_{18}N_2$	226.3	3.2
884.	Chaetoglobosine D	$C_{32}H_{36}N_2O_5$	528.7	3.2
885.	Chromomycin A2	$C_{59}H_{86}O_{26}$	1211.3	3.2
886.	Chrysazin	$C_{14}H_8O_4$	240.2	3.2
887.	Costaclavine	$C_{16}H_{20}N_2$	240.4	3.2
888.	Desertorin B	$C_{23}H_{20}O_8$	424.4	3.2
889.	Erythroglaucin	$C_{12}H_{16}O_6$	300.3	3.2
890.	Festuclavine	$C_{16}H_{20}N_2$	240.4	3.2
891.	Fusarin A	$C_{23}H_{29}NO_6$	415.5	3.2
892.	Homodestruxin B	$C_{31}H_{53}N_5O_7$	607.8	3.2
893.	Javanicin D	$C_{32}H_{40}O_{12}$	616.7	3.2
894.	Meleagrin C	$C_{29}H_{33}N_5O_5$	531.6	3.2
895.	Mitorubrin	$C_{21}H_{18}O_7$	382.4	3.2
896.	Mycophenolic acid	$C_{17}H_{20}O_6$	320.3	3.2
897.	Luteusin A	$C_{19}H_{23}ClO_4$	350.8	3.2
898.	Paspalinin	$C_{27}H_{31}NO_4$	433.6	3.2
899.	Pebrolide	$C_{24}H_{30}O_7$	430.5	3.2
900.	Penigequinolone A	$C_{27}H_{33}NO_6$	467.6	3.2
901.	Penigequinolone B	$C_{27}H_{33}NO_6$	467.6	3.2
902.	Phomol	$C_{22}H_{36}O_7$	412.5	3.2
903.	Preechinulin	$C_{19}H_{23}N_3O_2$	325.4	3.2
904.	Pyroclavine	$C_{16}H_{20}N_2$	240.4	3.2
905.	Roquefortine F	$C_{23}H_{25}N_5O_3$	419.5	3.2
906.	Rubrocristin	$C_{16}H_{12}O_6$	300.3	3.2
907.	Stemphone	$C_{30}H_{42}O_8$	530.7	3.2
908.	Stemphone E	$C_{30}H_{44}O_9$	548.7	3.2
909.	Aspergillusene C	$C_{15}H_{20}O_4$	264.3	3.3
910.	Austalide A	$C_{28}H_{36}O_9$	516.6	3.3
911.	Austalide G	$C_{28}H_{38}O_9$	518.6	3.3
912.	Austalide H	$C_{26}H_{36}O_8$	476.6	3.3
913.	Austalide L	$C_{25}H_{32}O_6$	428.5	3.3
914.	Carboxyfusarin C	$C_{22}H_{27}NO_7$	417.5	3.3
915.	Chaetomugilin D	$C_{23}H_{27}ClO_6$	434.9	3.3

916.	Chaetomugilin S	C23H27ClO6	434.9	3.3
917.	Chaetoviridin A	C23H25ClO6	432.9	3.3
918.	Chaetoviridin C	C23H27ClO6	434.9	3.3
919.	Cladosporin	C16H20O5	292.3	3.3
920.	Cladospirone F	C20H14O5	334.3	3.3
921.	Corynesidone B	C16H14O5	286.3	3.3
922.	Culmorin	C15H26O2	238.4	3.3
923.	Cytochalasin A	C29H35NO5	477.6	3.3
924.	Decaspirone I	C20H16O4	320.3	3.3
925.	5'-Epichaetoviridin A	C23H25ClO6	432.9	3.3
926.	Falconensin E	C23H25ClO7	448.9	3.3
927.	Geosmin	C12H22O	182.3	3.3
928.	Gregatin A	C16H20O4	276.3	3.3
929.	Helminthosporin	C15H10O5	270.2	3.3
930.	Islandicin	C15H10O5	270.2	3.3
931.	Isocladosporin	C16H20O5	292.3	3.3
932.	Isoemodin	C15H10O5	270.2	3.3
933.	5-Methoxysterigmatocystin	C19H14O7	354.3	3.3
934.	Monacolin X	C24H34O6	418.5	3.3
935.	Nalgiolaxin	C18H15ClO6	362.8	3.3
936.	Neoechinulin A	C19H21N3O2	323.4	3.3
937.	Ent-Neoechinulin A	C19H21N3O2	323.4	3.3
938.	Pyripyropene Q	C30H37NO8	539.6	3.3
939.	Rubrofusarin B	C16H14O5	286.3	3.3
940.	Stachybotrin D	C26H35NO5	441.6	3.3
941.	Stachybotrin E	C29H39NO8	529.6	3.3
942.	Stachybotrin F	C29H39NO8	529.6	3.3
943.	Torreyol	C15H26O	222.4	3.3
944.	Xanalteric acid I	C20H12O7	364.3	3.3
945.	Aurantionone	C20H20O8	388.4	3.4
946.	Aurofusarin	C30H18O12	570.5	3.4
947.	Bikaverin	C20H14O8	382.3	3.4
948.	Brevianamide P	C22H23N3O3	377.4	3.4
949.	Brevianamide R	C22H25N3O3	379.5	3.4
950.	Brevicompanine G	C23H29N3O3	395.5	3.4
951.	Communesin A	C28H32N4O2	456.6	3.4
952.	Cytochalasin B	C29H37NO5	479.6	3.4

953.	Cytochalasin G	C29H34N2O4	474.3	3.4
954.	Cytochalasin H	C30H39NO5	493.6	3.4
955.	Deoxaphomin	C29H37NO4	463.6	3.4
956.	Emestrin I	C29H30N2O7S2	582.7	3.4
957.	Mollisin	C14H10Cl2O4	313.1	3.4
958.	Monorden	C18H17ClO6	364.8	3.4
959.	Ochratoxin alpha	C11H9ClO5	256.6	3.4
960.	Ophiobolin J	C25H36O4	400.6	3.4
961.	Ophiobolin methoxy bromide	C26H39BrO5	511.5	3.4
962.	Palmarumycin BG1	C20H14O5	334.3	3.4
963.	Penochalasin E	C32H38N2O5	530.7	3.4
964.	Penochalasin F	C32H38N2O5	530.7	3.4
965.	Pyripyropene J	C33H41NO10	611.7	3.4
966.	Pyripyropene K	C33H41NO10	611.7	3.4
967.	Pyripyropene L	C33H41NO10	611.7	3.4
968.	Radicicol	C18H17ClO6	364.8	3.4
969.	Stachybotrin G	C28H38N2O5	482.6	3.4
970.	Stephacidin A	C26H29N3O3	431.5	3.4
971.	Sterigmatocystin	C18H12O6	324.3	3.4
972.	Stemphone D	C30H42O8	530.7	3.4
973.	Tryprostatin A	C22H27N3O3	381.5	3.4
974.	Yanuthone F	C22H32O4	360.5	3.4
975.	AF toxin III	C22H32O8	424.5	3.5
976.	AF Toxin IIIc	C22H32O8	424.5	3.5
977.	AF Toxin IIIb	C22H32O8	424.5	3.5
978.	Arisugacin D	C29H36O8	512.6	3.5
979.	Aspochalasin J	C24H35NO4	401.6	3.5
980.	Deoxaphomin B	C29H37NO4	463.6	3.5
981.	Deoxybrevianamide E	C21H25N3O2	351.5	3.5
982.	Chaetomugilin L	C23H29ClO5	420.9	3.5
983.	Chrysophanol	C15H10O4	254.2	3.5
984.	Cytochalasin E	C28H33NO7	495.6	3.5
985.	Fiscalin B	C23H22N4O2	386.5	3.5
986.	Monacolin L carboxylate	C19H29O4	321.4	3.5
987.	Memnobotrin B	C27H37NO6	471.6	3.5
988.	Ophiobolin A	C25H36O4	400.6	3.5
989.	6-Epi-ohpiobolin A	C25H36O4	400.6	3.5

990.	Palmarumycin C3	C20H12O6	348.3	3.5
991.	Paspalitrem B	C32H39NO5	517.7	3.5
992.	Paxilline	C27H33NO4	435.6	3.5
993.	Penitrem E	C37H45NO6	599.8	3.5
994.	Petrosifungin A	C33H47N5O6	609.8	3.5
995.	Phenylpyropene A	C32H38O10	582.7	3.5
996.	Pyripyropene G	C27H33NO6	467.6	3.5
997.	Topopyrone A	C18H9ClO7	372.7	3.5
998.	Tryprostatin B	C21H25N3O2	351.5	3.5
999.	Xantocillin X methyl ether	C19H14N2O2	302.3	3.5
1000.	Alterporriol X	C32H26O11	586.5	3.6
1001.	Austocystin B	C22H20O7	396.4	3.6
1002.	Brevianamide V	C21H23N3O2	349.4	3.6
1003.	Cercophorin C	C20H16O10	416.3	3.6
1004.	Chaetoglobosine U	C32H36N2O5	528.7	3.6
1005.	Desertorin C	C24H22O8	438.4	3.6
1006.	Falconensin M	C22H22Cl2O7	469.3	3.6
1007.	Monacolin L	C19H28O3	304.4	3.6
1008.	Monacolin L lactone	C19H28O3	304.4	3.6
1009.	Neoechinulin B	C19H19N3O2	321.4	3.6
1010.	Ophiobolin B	C25H38O4	402.6	3.6
1011.	Penitrem G	C37H45NO5	583.8	3.6
1012.	Solanapyrone C	C19H25NO4	331.4	3.6
1013.	Solanapyrone G	C17H21NO3	287.4	3.6
1014.	Solistatin	C18H20O3	284.4	3.6
1015.	Sorbicillin	C14H16O3	232.3	3.6
1016.	Zearalenone	C18H22O5	318.4	3.6
1017.	19-O-Acetylchaetoglobosin D	C34H38N2O6	570.7	3.7
1018.	Arisugacin C	C27H32O6	452.6	3.7
1019.	Atpenin A5	C15H21Cl2NO5	366.2	3.7
1020.	Atranone D	C24H34O4	386.5	3.7
1021.	Atranone E	C24H34O4	386.5	3.7
1022.	Austalide P	C26H36O7	460.6	3.7
1023.	Brevianamide K	C21H21N3O2	347.4	3.7
1024.	Chaetoglobosine A	C32H36N2O5	528.7	3.7
1025.	Chaetoglobosine C	C32H36N2O5	528.7	3.7
1026.	Chaetomugilin H	C22H27ClO4	390.9	3.7

1027.	Fumonisin A2	C36H61NO15	747.9	3.7
1028.	Fusarielin D	C25H36O4	400.6	3.7
1029.	Griseophenone B	C16H15ClO6	338.7	3.7
1030.	Ophiobolin L	C25H36O5	416.6	3.7
1031.	6-Epi-Ophiobolin L	C25H36O5	416.6	3.7
1032.	Phomazarin	C19H17NO8	387.3	3.7
1033.	Solanapyrone A	C18H22O4	302.4	3.7
1034.	Solanapyrone B	C18H24O4	304.4	3.7
1035.	Solanapyrone D	C18H22O4	302.4	3.7
1036.	Solanapyrone E	C18H24O4	304.4	3.7
1037.	Stachibotrilactam	C23H31NO4	385.5	3.7
1038.	Vermixocin A	C21H24O6	372.4	3.7
1039.	Vinblastine	C46H58N4O9	811.0	3.7
1040.	Andrastin A	C28H38O7	486.6	3.8
1041.	Austalide I	C27H34O8	486.6	3.8
1042.	Austocystin A	C19H13ClO6	372.8	3.8
1043.	2-Bromo-Alpha-Ergocryptine	C32H40BrN5O5	656.6	3.8
1044.	Candidusin B	C20H16O7	368.3	3.8
1045.	Chaetomugilin E	C24H29ClO6	448.9	3.8
1046.	Chaetoxanthone B	C20H18O6	354.4	3.8
1047.	Cyclopiazonic acid	C20H20N2O3	336.4	3.8
1048.	Cytoglobosin D	C32H38N2O4	514.7	3.8
1049.	Ergocristam	C35H39N5O4	593.7	3.8
1050.	Falconensin N	C22H24Cl2O7	471.3	3.8
1051.	Fructigenine B	C24H31N3O3	409.5	3.8
1052.	Fumitremorgin A	C32H41N3O7	579.7	3.8
1053.	Janthitrem B	C37H47NO5	585.8	3.8
1054.	Ochratoxin hydroquinone	C20H19NO7	385.4	3.8
1055.	Penitrem A	C37H44ClNO6	634.2	3.8
1056.	Penostatin G	C23H34O5	390.5	3.8
1057.	Penostatin H	C23H34O5	390.5	3.8
1058.	Phomapyrone A	C14H18O3	234.3	3.8
1059.	Phomenin B	C14H18O3	234.3	3.8
1060.	Pyripyropene N	C31H39NO8	553.7	3.8
1061.	Rosenonolactone	C20H28O3	316.4	3.8
1062.	Rubellin A	C30H22O9	526.5	3.8
1063.	Rubellin C	C30H22O9	526.5	3.8

1064.	Thomitrem E	C37H45NO6	599.8	3.8
1065.	Xanthocillin X dimethyl ether	C20H16N2O2	316.4	3.8
1066.	Verrucofortine	C24H31N3O3	409.5	3.8
1067.	Yanuthone D	C28H38O8	502.6	3.8
1068.	Aspergiolide C	C29H16O10	524.4	3.9
1069.	Austocystin C	C23H22O7	410.4	3.9
1070.	Austocystin H	C22H18O7	394.4	3.9
1071.	Brevicompanine H	C24H31N3O3	409.5	3.9
1072.	Communesin G	C29H34N4O2	470.6	3.9
1073.	Compactin (Mevastatin)	C23H34O5	390.5	3.9
1074.	Fusarielin A	C25H38O4	402.6	3.9
1075.	31-epi-Lolitrem N	C37H49NO7	619.8	3.9
1076.	Lolitrem J	C39H51NO8	661.8	3.9
1077.	Lolitrem N	C37H49NO7	619.8	3.9
1078.	Lolitriol	C37H49NO7	619.8	3.9
1079.	Monacolin N	C19H28O3	304.4	3.9
1080.	Mycelianamide	C22H28N2O5	400.5	3.9
1081.	Ophiobolin I	C25H36O3	384.6	3.9
1082.	Pachybasin	C15H10O3	238.2	3.9
1083.	Palmarumycin P5	C21H16O5	348.4	3.9
1084.	Penostatin D	C22H34O3	346.5	3.9
1085.	Pyripyropene I	C34H43NO10	625.7	3.9
1086.	Scytalidin	C22H28O7	404.5	3.9
1087.	Tricycloalternarene 4a	C22H32O3	344.5	3.9
1088.	Zearalanone	C18H24O5	320.4	3.9
1089.	Yanuthone M	C24H36O5	404.6	3.9
1090.	Andrastin B	C28H40O7	488.6	4.0
1091.	Arugosin D	C25H28O7	440.5	4.0
1092.	Arisugacin E	C27H34O6	454.6	4.0
1093.	Aspulvinone F	C27H28O7	464.5	4.0
1094.	Averantin	C20H20O7	372.4	4.0
1095.	Cercosporin	C29H26O10	534.5	4.0
1096.	Chaetoglobosine F	C32H38N2O5	530.7	4.0
1097.	Cycloaspeptide D	C35H41N5O6	627.7	4.0
1098.	Dihydrosorbicillin	C14H18O3	234.3	4.0
1099.	Dihydroxyaflavinine	C28H39NO3	437.6	4.0
1100.	Dithiosilvatin	C18H22N2O3S2	378.5	4.0

1101.	Falconensin A	C23H24Cl2O7	483.3	4.0
1102.	Fumagillin	C26H34O7	458.6	4.0
1103.	Fusarin	C22H29NO5	387.5	4.0
1104.	Memnobotrin A	C25H33NO5	427.5	4.0
1105.	Palmarumycin CP17	C20H14O5	334.3	4.0
1106.	Penochalasin B	C32H35N3O3	509.7	4.0
1107.	Penochalasin G	C32H38N2O4	514.7	4.0
1108.	Pyripyropene H	C28H35NO6	481.5	4.0
1109.	Pyripyropene M	C32H39NO9	581.6	4.0
1110.	Pyripyropene O	C29H35NO7	509.6	4.0
1111.	Rubellin B	C30H22O10	542.5	4.0
1112.	Rubellin D	C30H22O10	542.5	4.0
1113.	Terpendole M	C32H41NO6	535.7	4.0
1114.	Zearalenol alpha	C18H24O5	320.4	4.0
1115.	Zearalenol beta	C18H24O5	320.4	4.0
1116.	Xanthomegnine	C30H22O12	574.5	4.0
1117.	Yanuthone A	C24H34O5	402.5	4.0
1118.	Epi-Yanuthone A	C24H34O5	402.5	4.0
1119.	Yanuthone C	C24H34O5	402.5	4.0
1120.	Epi-Yanuthone C	C24H34O5	402.5	4.0
1121.	Yanuthone L	C24H34O5	402.5	4.0
1122.	Apicidin A	C33H47N5O5	593.8	4.1
1123.	Apicidin B	C33H47N5O6	609.8	4.1
1124.	Apicidin C	C33H47N5O6	609.8	4.1
1125.	Apicidin F	C35H43N5O7	645.8	4.1
1126.	Aspergillusene A	C15H22O2	234.3	4.1
1127.	Atrovenetin	C19H18O6	342.3	4.1
1128.	Austalide Q	C26H34O7	458.6	4.1
1129.	Brevicompanine C	C21H27N3O2	353.5	4.1
1130.	Brevione C	C27H32O4	420.6	4.1
1131.	Canescin (Deserpidin)	C32H38N2O8	578.7	4.1
1132.	Cercophorin B	C20H16O10	416.3	4.1
1133.	Emestrin K	C29H30N2O6S2	566.7	4.1
1134.	Falconensin B	C23H26Cl2O7	485.3	4.1
1135.	Fructigenine A (Puberulin)	C27H29N3O3	443.6	4.1
1136.	Fungisporin	C28H36N4O4	492.6	4.1
1137.	Norsolorinic acid	C20H18O7	370.4	4.1

1138.	Ochratoxin B	C20H19NO6	369.4	4.1
1139.	Penostatin F	C22H32O3	344.5	4.1
1140.	Penostatin I	C22H32O3	344.5	4.1
1141.	Stemphone G	C30H42O9	546.7	4.1
1142.	Terphenyllin	C20H18O5	338.4	4.1
1143.	Trachyspic acid dimethylester	C23H34O9	454.5	4.1
1144.	Valyl-ochratoxin A	C16H18ClNO6	355.8	4.1
1145.	Variecoxanthone A	C20H20O5	340.4	4.1
1146.	Altersetin	C23H31NO3	369.5	4.2
1147.	Apicidin K	C35H45N5O6	631.8	4.2
1148.	Aspergiolide A	C25H16O9	460.4	4.2
1149.	Aspergiolide D	C30H18O10	538.5	4.2
1150.	Aspergillusene B	C15H18O3	246.3	4.2
1151.	Aurasperone 8-methyl ether	C30H26O12	578.5	4.2
1152.	Aurasperone C	C31H28O12	592.6	4.2
1153.	Brevianamide T	C21H19N3O2	345.4	4.2
1154.	Calfostin D	C30H30O10	550.6	4.2
1155.	Candidusin A	C20H16O6	352.3	4.2
1156.	Chaetomugilin F	C23H25ClO5	416.9	4.2
1157.	Chaetoviridin E	C23H23ClO5	414.8	4.2
1158.	Falconensin H	C22H18Cl2O7	465.3	4.2
1159.	Fumonisin FP2	C39H62NO15	784.9	4.2
1160.	Communesin D	C32H34N4O3	522.7	4.2
1161.	Janthitrem G	C39H51NO6	629.8	4.2
1162.	Ophiobolin M	C25H36O3	384.6	4.2
1163.	Stachybotrin B	C23H31NO4	385.5	4.2
1164.	19-O-Acetylchaetoglobosin A	C34H38N2O6	570.7	4.3
1165.	Alpha-Zearalanol	C18H26O5	322.4	4.3
1166.	Andrastin D	C26H36O5	428.6	4.3
1167.	Anomalin	C24H26O7	426.5	4.3
1168.	Apicidin J	C34H40N5O7	630.7	4.3
1169.	Avenaciolid	C15H22O4	266.3	4.3
1170.	Austalide K	C25H32O5	412.5	4.3
1171.	Beta-Zearalanol	C18H26O5	322.4	4.3
1172.	Brevicompanine E	C25H33N3O3	423.6	4.3
1173.	Chaetoglobosine J	C32H36N2O4	512.7	4.3
1174.	Chaetoviridin G	C23H25ClO5	416.9	4.3

1175.	Communesin H	C30H36N4O2	484.6	4.3
1176.	Cycloaspeptide A	C36H43N5O6	641.8	4.3
1177.	Hymenosetin	C23H33NO4	387.5	4.3
1178.	Monacolin K (Lovastatin)	C24H36O5	404.6	4.3
1179.	ent-Penostatin A	C22H32O3	344.5	4.3
1180.	Stemphone C	C30H42O7	514.7	4.3
1181.	Vermixocin B	C23H26O7	414.5	4.3
1182.	Apicidin	C34H49N5O6	623.8	4.4
1183.	Asperglaucide	C27H28N2O4	444.5	4.4
1184.	Asperglaucide	C27H28N2O4	444.5	4.4
1185.	Brevicompanine A	C22H29N3O2	367.5	4.4
1186.	Brevicompanine B	C22H29N3O2	367.5	4.4
1187.	allo-Brevicompanine A	C22H29N3O2	367.5	4.4
1188.	allo-Brevicompanine B	C22H29N3O2	367.5	4.4
1189.	Chaetoglobosine K	C34H40N2O5	556.7	4.4
1190.	Chaetoglobosine M	C34H40N2O5	556.7	4.4
1191.	Chaetoviridin F	C23H25ClO5	416.9	4.4
1192.	Chaetoxanthone C	C20H19ClO6	390.8	4.4
1193.	4'-Epichaetoviridin F	C23H25ClO5	416.9	4.4
1194.	Fumonisin FP3	C39H62NO15	784.9	4.4
1195.	Fusaproliferin	C27H40O5	444.6	4.4
1196.	Lolilline	C37H47NO6	601.8	4.4
1197.	Ochratoxin B methyl ester	C21H21NO6	383.4	4.4
1198.	Ochratoxin TA	C20H18ClNO7	419.8	4.4
1199.	Palmarumycin CP2	C20H14O4	318.3	4.4
1200.	Pyripyropene P	C30H37NO7	523.6	4.4
1201.	Pyripyropene R	C30H37NO7	523.6	4.4
1202.	Solanapyrone K	C21H29NO4	359.5	4.4
1203.	Solanapyrone L	C19H25NO3	315.4	4.4
1204.	Thomitrem A	C37H44ClNO6	634.2	4.4
1205.	Tricycloalternarene 5a	C22H32O3	344.5	4.4
1206.	Yanuthone X2	C22H32O4	360.5	4.4
1207.	Anhydroepiophiobolin A	C25H34O3	382.5	4.5
1208.	Anicequol	C30H48O6	504.7	4.5
1209.	Aspergillide D	C40H49N5O8	727.9	4.5
1210.	Aspergiolide B	C26H18O9	474.4	4.5
1211.	Brevicompanine D	C24H33N3O3	411.6	4.5

1212.	Brevione B	C27H36O4	424.6	4.5
1213.	Candidusin C	C21H18O6	366.4	4.5
1214.	Falconensin C	C25H26Cl2O8	525.4	4.5
1215.	Fusarielin C	C25H38O3	386.6	4.5
1216.	16-O-Deacetylfusidic acid	C28H44O5	460.7	4.5
1217.	Nigerapyrone A	C20H18O3	306.4	4.5
1218.	Penitrem B	C37H45NO5	583.8	4.5
1219.	Penochalasin D	C32H37N3O3	511.7	4.5
1220.	Penostatin J	C22H30O3	342.5	4.5
1221.	Ophiobolin A lacton	C25H36O4	400.6	4.5
1222.	Solanapyrone J	C20H26O4	330.4	4.5
1223.	Solanapyrone M	C20H28O4	332.4	4.5
1224.	Stephacidin B	C52H54N6O8	891.0	4.5
1225.	Terpendole G	C28H37NO4	451.6	4.5
1226.	Terpendole J	C32H43NO5	521.7	4.5
1227.	Tricycloalternarene 4b	C22H32O3	344.5	4.5
1228.	Aspulvinone I	C22H20O5	364.4	4.6
1229.	Aurasperone B	C32H30O12	606.6	4.6
1230.	Brevicompanine F	C26H35N3O3	437.6	4.6
1231.	Chaetoglobosine 542	C34H42N2O4	542.7	4.6
1232.	Cladosporide B	C25H38O3	386.6	4.6
1233.	Cladosporide D	C25H38O3	386.6	4.6
1234.	Cytochalasin F6	C29H37NO5	479.6	4.6
1235.	Meleagrin D	C32H37N5O5	571.7	4.6
1236.	Ophiobolin B lactone	C25H38O4	402.6	4.6
1237.	Palmarumycin CP1	C20H12O4	316.3	4.6
1238.	Paspalicine	C27H31NO3	417.5	4.6
1239.	Staplabin	C28H39NO6	485.6	4.6
1240.	Terpendole F	C28H39NO4	453.6	4.6
1241.	Arugosin H	C20H20O6	356.4	4.7
1242.	Bafilomycin E	C44H65NO13	816.0	4.7
1243.	Brevione A	C27H34O4	422.6	4.7
1244.	Cycloaspeptide E	C36H43N5O5	625.8	4.7
1245.	Falconensin D	C25H28Cl2O8	527.4	4.7
1246.	Fumigaclavine C	C23H30N2O2	366.5	4.7
1247.	Lolitrem A	C42H55NO8	701.9	4.7
1248.	Lolitrem K	C37H47NO6	601.8	4.7

1249.	Ochratoxin A	C20H18ClNO6	403.8	4.7
1250.	Ophiobolin H	C25H38O3	386.6	4.7
1251.	Penitrem D	C37H45NO4	567.8	4.7
1252.	Phomasetin	C25H35NO4	413.6	4.7
1253.	Variecoxanthone C	C25H28O6	424.5	4.7
1254.	Terpendole B	C27H35NO3	421.6	4.7
1255.	Arisugacin G	C27H32O5	436.6	4.8
1256.	Chaetomugilin K	C23H29ClO4	404.9	4.8
1257.	Communesin C	C31H34N4O2	494.6	4.8
1258.	Communesin F	C28H32N4O	440.6	4.8
1259.	Nigerapyrone B	C21H20O3	320.3	4.8
1260.	Penitrem F	C37H44ClNO5	618.2	4.8
1261.	Penostatin E	C22H32O3	344.5	4.8
1262.	Ochratoxin A methyl ester	C21H20ClNO6	417.8	4.8
1263.	Ochratoxin B ethyl ester	C22H23NO6	397.4	4.8
1264.	Secopenitrem D	C37H47NO4	569.8	4.8
1265.	Yanuthone B	C24H32O5	400.5	4.8
1266.	Apicidin D2	C34H51N5O6	625.8	4.9
1267.	Apicidin D3	C34H51N5O6	625.8	4.9
1268.	Aspergilone A	C26H26O3	386.5	4.9
1269.	Aspulvinone C	C27H28O6	448.5	4.9
1270.	Chaetoglobosin T	C32H38N2O3	498.7	4.9
1271.	Cladosporide A	C25H40O3	388.6	4.9
1272.	Cladosporide C	C25H40O3	388.6	4.9
1273.	Communesin B	C32H36N4O2	508.7	4.9
1274.	Deoxypaxillin	C27H33NO3	419.6	4.9
1275.	Ochratoxin A ethylamide	C22H23ClN2O5	430.9	4.9
1276.	Oligomycin E	C45H72O13	821.1	4.9
1277.	Ophiobolin K	C25H36O3	384.6	4.9
1278.	6-Epi-Ophiobolin K	C25H36O3	384.6	4.9
1279.	Paspaline B	C28H37NO3	435.6	4.9
1280.	Siccanine	C22H30O3	342.5	4.9
1281.	Terpendole C	C32H41NO5	519.7	4.9
1282.	Tricycloalternarene 5b	C22H32O3	344.5	4.9
1283.	Arugosin C	C25H28O6	424.5	5.0
1284.	Aspochalasin Z	C24H35NO2	369.6	5.0
1285.	3,11-Diketofusidic acid	C31H44O6	512.6	5.0

1286.	Equisetin	C22H31NO4	373.5	5.0
1287.	Penitrem C	C37H44ClNO4	602.2	5.0
1288.	Phenylpyropene B	C30H36O7	508.6	5.0
1289.	Roquefortine E	C27H31N5O2	457.6	5.0
1290.	Yanuthone X1	C24H34O5	402.5	5.0
1291.	Aflatrem	C32H39NO4	501.7	5.1
1292.	Arisugacin F	C27H34O5	438.6	5.1
1293.	Aurasperone F	C31H26O11	574.5	5.1
1294.	Byssochlamysol	C32H50O7	546.7	5.1
1295.	Cladofulvin	C30H18O10	538.5	5.1
1296.	9-Deacetoxyfumigaclavine C	C21H28N2	308.5	5.1
1297.	Fumigacin (Helvolic acid)	C33H44O8	568.7	5.1
1298.	Fusarielin J	C33H46O6	538.7	5.1
1299.	Lyclavatol	C28H50O4	450.7	5.1
1300.	Ochratoxin Tc	C22H22ClNO7	447.9	5.1
1301.	Paspalitrem A	C32H39NO4	501.7	5.1
1302.	Paspalitrem C	C32H39NO4	501.7	5.1
1303.	Skyrin	C30H18O10	538.5	5.1
1304.	Andrastin C	C28H40O6	472.6	5.2
1305.	Aristolochene	C15H24	204.4	5.2
1306.	Chaetoglobosin 540	C34H40N2O4	540.7	5.2
1307.	Deoxaphomin C	C29H37NO3	447.6	5.2
1308.	Ophiobolin O	C27H42O4	430.6	5.2
1309.	Semicochliodinol A	C27H22N2O4	438.5	5.2
1310.	Semicochliodinol B	C27H22N2O4	438.5	5.2
1311.	Viomellein	C30H24O11	560.5	5.2
1312.	Yanuthone K	C24H34O4	386.5	5.2
1313.	Aspulvinone A	C27H28O5	432.5	5.3
1314.	Isoechinulin A	C24H29N3O2	391.5	5.3
1315.	11-Ketofusidic acid	C31H46O6	514.7	5.3
1316.	Neoechinulin D	C24H29N3O2	391.5	5.3
1317.	Nigerapyrone F	C20H20O3	308.4	5.3
1318.	Nigerapyrone G	C20H20O3	308.4	5.3
1319.	Nigerapyrone H	C20H20O3	308.4	5.3
1320.	Ophiobolin C	C25H38O3	386.6	5.3
1321.	Penostatin C	C22H30O2	326.5	5.3
1322.	Verrucosine A	C25H40O5	420.6	5.3

1323.	Verrucosine B	C25H40O5	420.6	5.3
1324.	Terpendole E	C28H39NO3	437.6	5.3
1325.	Aurasperone E	C32H28O11	588.6	5.4
1326.	Chaetochromin C	C29H24O10	532.5	5.4
1327.	Fusapyrone	C34H54O9	606.8	5.4
1328.	Lolitrem E	C42H57NO7	687.9	5.4
1329.	Ochratoxin A ethyl ester	C22H22ClNO6	431.9	5.4
1330.	Ochratoxin C	C22H22ClNO6	431.9	5.4
1331.	Aspulvinone B1	C27H26O6	446.5	5.5
1332.	Aspulvinone D	C27H28O6	448.5	5.5
1333.	Fusarielin L	C28H42O4	442.6	5.5
1334.	Fusidic acid	C31H48O6	516.7	5.5
1335.	Neoechinulin C	C24H27N3O2	389.5	5.5
1336.	Aspochalamin A	C44H55N5O6	750.0	5.6
1337.	Cerevisterol	C28H46O3	430.7	5.6
1338.	Enniatin J1	C31H53N3O9	611.8	5.6
1339.	Chaetochalasin A	C27H39NO2	409.6	5.7
1340.	Sambutoxin	C28H39NO4	453.6	5.7
1341.	Alterporriol Q	C32H22O10	566.5	5.8
1342.	Bafilomycin B2	C45H67NO13	830.0	5.8
1343.	Bafilomycin D	C35H56O8	604.8	5.8
1344.	Chaetochromin D	C30H24O10	544.5	5.8
1345.	Lolitrem B	C42H55NO7	685.9	5.8
1346.	31-epi-Lolitrem B	C42H55NO7	685.9	5.8
1347.	Lolitrem F	C42H55NO7	685.9	5.8
1348.	31-epi-Lolitrem F	C42H55NO7	685.9	5.8
1349.	Ophiobolin D	C25H36O4	400.6	5.8
1350.	Ophiobolin G	C25H34O2	366.5	5.8
1351.	6-Epi-Ophiobolin G	C25H34O2	366.5	5.8
1352.	Arugosin A	C25H28O6	424.5	5.9
1353.	Arugosin B	C25H28O6	424.5	5.9
1354.	Arugosin K	C28H32O7	480.6	5.9
1355.	Aspulvinone A	C27H28O5	432.5	5.9
1356.	Bafilomycin B1	C44H65NO13	816.0	5.9
1357.	Chaetochromin A	C30H26O10	546.5	5.9
1358.	Chaetochromin B	C30H26O10	546.5	5.9
1359.	Emindole DB	C28H39NO2	421.6	5.9

1360.	Rubrosulphin	C29H20O10	528.5	5.9
1361.	Shamixanthone	C25H26O5	406.5	5.9
1362.	Triterpenoid	C30H48O7S	552.8	5.9
1363.	Aurasperone D	C31H24O10	556.5	6.0
1364.	Bafilomycin A1	C35H58O9	622.8	6.0
1365.	Bafilomycin A2	C36H60O9	636.9	6.0
1366.	Bafilomycin F	C42H65NO13S	824.0	6.0
1367.	Emindole SB beta-D-mannoside	C34H49NO6	567.8	6.0
1368.	Isoemericellin	C25H28O5	408.5	6.0
1369.	Lolitrem C	C42H57NO7	687.9	6.0
1370.	Retigeric acid B	C30H46O6	502.7	6
1371.	Variecoxanthone B	C25H28O5	408.5	6.0
1372.	9,11-Anhydrofusidic Acid	C31H46O5	498.7	6.1
1373.	Aspergilone B	C39H40O6	604.7	6.1
1374.	Asperphenamate	C32H30N2O4	506.6	6.1
1375.	Enniatin K1	C32H55N3O9	625.8	6.1
1376.	Enniatin L	C34H59N3O10	669.9	6.1
1377.	Oligomycin B	C45H72O12	805.1	6.1
1378.	Prenylcandidusin A	C25H24O6	420.5	6.2
1379.	Bafilomycin K	C35H58O8	606.8	6.2
1380.	Brevianamide S	C42H40N6O4	692.8	6.2
1381.	Aureobasidin S1	C59H90N8O12	1135.5	6.3
1382.	Aurasperone A	C32H26O10	570.6	6.3
1383.	Bafilomycin C2	C40H62O12	734.9	6.3
1384.	Brevianamide J	C42H42N6O5	710.8	6.3
1385.	Paspaline	C28H39NO2	421.6	6.3
1386.	Vioxanthin	C30H26O10	546.5	6.3
1387.	Bafilomycin C1	C39H60O12	720.9	6.4
1388.	Calphostin B	C37H34O11	654.7	6.4
1389.	Enniatin B2	C32H55N3O9	625.8	6.4
1390.	Prenylcandidusin C	C26H26O6	434.5	6.4
1391.	Aspulvinone H	C27H28O5	432.5	6.5
1392.	Bafilomycin H	C37H62O9	650.9	6.5
1393.	Bafilomycin V1	C37H64O10	668.9	6.5
1394.	Citreomontanin	C23H28O3	352.5	6.5
1395.	Enniatin B	C33H57N3O9	639.8	6.5
1396.	Aflavinine	C28H39NO	405.6	6.6

1397.	Apicidin E	C34H51N5O5	609.8	6.6
1398.	Aspochalamin C	C44H55N5O5	734.0	6.6
1399.	Bafilomycin G	C36H60O9	636.9	6.6
1400.	Deoxyfusapyrone	C34H54O8	590.8	6.6
1401.	Nonactin	C40H64O12	736.9	6.6
1402.	Oligomycin D	C44H72O11	777.1	6.6
1403.	Flavoglaucin	C19H28O3	304.4	6.7
1404.	Fusarielin K	C36H50O6	578.8	6.7
1405.	Prenylcandidusin B	C27H28O6	448.5	6.7
1406.	Beauvenniatin C	C37H57N3O10	703.9	6.8
1407.	Nidulin	C20H17Cl3O5	443.7	6.8
1408.	Chaetoglobosin 510	C34H42N2O2	510.7	6.9
1409.	Cyclosporin C	C62H111N11O13	1218.6	6.9
1410.	Enniatin B1	C34H59N3O9	653.9	6.9
1411.	Enniatin D	C34H59N3O9	653.9	6.9
1412.	Enniatin N	C36H63N3O10	697.9	6.9
1413.	Lichesterol	C28H44O	396.7	6.9
1414.	Cyclosporin B	C61H109N11O12	1188.6	7.0
1415.	Echinulin	C29H39N3O2	461.7	7.0
1416.	Beauvericin G3	C42H51N3O9	741.9	7.1
1417.	Cochliodinol	C32H30N2O4	506.6	7.1
1418.	Neocochliodinol	C32H30N2O4	506.6	7.1
1419.	Oligomycin A	C45H74O10	775.1	7.1
1420.	Ophiobolin F	C25H42O	358.6	7.1
1421.	Bafilomycin D1'	C39H62O11	706.9	7.2
1422.	Bafilomycin D2'	C40H64O11	720.9	7.2
1423.	Beauvenniatin B	C37H57N3O9	687.9	7.2
1424.	Emindole DA	C28H39NO	405.6	7.2
1425.	Monactin	C41H66O12	751.0	7.2
1426.	Nominine	C28H39NO	405.6	7.2
1427.	Aureobasidin S2b	C60H92N8O12	1117.4	7.3
1428.	Aureobasidin S3	C60H92N8O12	1117.4	7.3
1429.	Cyclosporin E	C61H109N11O12	1188.6	7.3
1430.	Cyclosporin L	C61H109N11O12	1188.6	7.3
1431.	Enniatin E	C36H63N3O10	697.9	7.3
1432.	Enniatin G	C35H61N3O9	667.9	7.3
1433.	Meleagrin B	C43H53N5O6	735.9	7.3

1434.	Retigeric acid A	C30H48O4	472.7	7.3
1435.	Stachybocin B	C52H70N2O11	899.1	7.3
1436.	Stachybocin C	C52H70N2O11	899.1	7.3
1437.	Aureobasidin S4	C60H92N8O12	1117.4	7.4
1438.	Beauvenniatin D	C41H57N3O10	751.9	7.4
1439.	Beauvenniatin E	C41H57N3O10	730.0	7.4
1440.	Emindole PB	C33H47NO2	489.7	7.4
1441.	Enniatin A1	C35H61N3O9	667.9	7.4
1442.	Ergosterol	C28H44O	396.7	7.4
1443.	Arugosin G	C29H34O6	478.6	7.5
1444.	Beauvericin G2	C43H53N3O9	755.9	7.5
1445.	Cyclosporin A	C62H111N11O12	1202.6	7.5
1446.	Cyclosporin H	C62H111N11O12	1202.6	7.5
1447.	Ionomycin	C41H72O9	709.0	7.5
1448.	Emindole Sb	C28H39NO	405.6	7.6
1449.	Enniatin C	C36H63N3O9	681.9	7.6
1450.	Aspochalamin D	C44H55N5O4	718.0	7.7
1451.	Bafilomycin I	C36H56O7	600.8	7.7
1452.	Dinactin	C42H68O12	765.0	7.7
1453.	Aureobasidin U2	C58H88N8O11	1073.4	7.8
1454.	Beauvenniatin A	C41H57N3O9	735.9	7.8
1455.	Enniatin A	C36H63N3O9	681.9	7.8
1456.	Enniatin MK 1688	C36H63N3O9	681.9	7.8
1457.	Calphostin I	C44H38O14	790.8	7.9
1458.	Aureobasidin E	C60H92N8O12	1117.4	8.0
1459.	Beauvericin G1	C44H55N3O9	769.9	8.0
1460.	Brassicasterol	C28H46O	398.7	8.0
1461.	Oligomycin C	C45H74O10	775.1	8.0
1462.	Aureobasidin J	C60H90N8O12	1115.4	8.1
1463.	Beauvericin J	C45H57N3O10	800.0	8.1
1464.	Emindole PA	C33H47NO2	489.7	8.1
1465.	Emindole PC	C33H47NO2	489.7	8.1
1466.	Aureobasidin B	C59H90N8O11	1087.4	8.2
1467.	Aureobasidin C	C59H90N8O11	1087.4	8.2
1468.	Aureobasidin D	C66H103N9O13	1230.6	8.2
1469.	Beauvericin D	C44H55N3O9	769.9	8.2
1470.	Beauvenniatin F	C40H63N3O9	730.0	8.2

1471.	Dihydrocyclosporin A	C62H113N11O12	1204.6	8.2
1472.	Aureobasidin F	C59H90N8O11	1087.4	8.3
1473.	Aureobasidin U1	C59H90N8O11	1087.4	8.3
1474.	Stachybocin A	C52H70N2O10	883.1	8.3
1475.	Aureobasidin T3	C59H90N8O11	1087.4	8.4
1476.	Beauvericin	C45H57N3O9	784.0	8.4
1477.	Aureobasidin A (Basifungin)	C60H92N8O11	1101.4	8.5
1478.	Aureobasidin I	C60H92N8O11	1101.4	8.5
1479.	Aureobasidin T1	C60H92N8O11	1101.4	8.5
1480.	Beauvericin H1	C45H56FN3O9	802.0	8.5
1481.	Dustanin	C30H52O2	444.7	8.5
1482.	Beauvericin H2	C45H55F2N3O9	819.9	8.6
1483.	Calphostin A	C44H38O12	758.8	8.6
1484.	Calphostin C	C44H38O14	790.8	8.6
1485.	Dihydrocyclosporin D	C63H115N11O12	1218.7	8.6
1486.	Beauvericin H3	C45H54F3N3O9	837.9	8.7
1487.	Beauvericin A	C46H59N3O9	798.0	8.8
1488.	22,23-Dihydrobrassicasterol	C28H48O	400.7	8.8
1489.	Aureobasidin T2	C61H94N8O11	1115.5	8.9
1490.	Aureobasidin T4	C61H94N8O11	1115.5	8.9
1491.	Aureobasidin L	C59H90N8O10	1071.4	9.0
1492.	Beauvericin B	C47H61N3O9	812.0	9.1
1493.	Valinomycin	C54H90N6O18	1111.3	9.1
1494.	Aureobasidin H	C59H90N8O10	1071.4	9.2
1495.	Aureobasidin M	C59H90N8O10	1071.4	9.2
1496.	Aureobasidin G	C60H92N8O10	1085.4	9.4
1497.	Aureobasidin K	C60H92N8O10	1085.4	9.4
1498.	Beauvericin C	C48H63N3O9	826.0	9.5
1499.	Marinomycin A	C58H76O14	997.2	10.0
1500.	Bassianolide	C48H84N4O12	909.2	10.1

*—Data were selected from PubChem database [57].

Table S2. Calculated partition coefficients of some polyaromatic hydrocarbons.*.

No.	Compound	Molecular formula	Molecular weight	XLog P3-AA
1.	Naphthalene	C10H8	128.1	3.3
2.	Acenaphthylene	C12H8	152.1	3.7
3.	Acenaphthene	C12H10	154.2	3.9
4.	Fluorene	C13H10	166.2	4.2
5.	Anthracene	C14H10	178.2	4.4
6.	Phenanthrene	C14H10	178.2	4.5
7.	Triphenilene	C18H12	228.2	4.9
8.	Pyrene	C16H10	286.3	4.9
9.	Fluoranthene	C16H10	202.2	5.2
10.	Chrysene	C18H12	228.2	5.7
11.	Benzo[c]phenanthrene	C18H12	228.2	5.7
12.	Benzo[a]fluorene	C17H12	216.2	5.7
13.	Benzo[c]fluorene	C17H12	216.2	5.7
14.	Benzo[b]fluorene	C17H12	216.2	5.8
15.	6-Methylbenz[a]anthracene	C19H14	242.3	5.8
16.	7,12-Dimethylbenz[a]anthracene	C20H16	256.3	5.8
17.	Perilene	C20H12	252.3	5.8
18.	Benz[a]anthracene	C18H12	228.2	5.9
19.	Naphthacene	C18H12	228.2	5.9
20.	Benzo[a]pyrene	C20H12	252.3	6.0
21.	7-Methylbenz[a]anthracene	C19H14	242.3	6.3
22.	6-Methylcholanthrene	C21H16	268.3	6.3
23.	Benzo[e]pyrene	C20H12	252.3	6.4
24.	7-Bromobenz[a]anthracene	C18H11Br	307.1	6.4
25.	Benzo[b]fluoranthene	C20H12	252.3	6.4
26.	Benzo[j]fluoranthene	C20H12	252.3	6.4
27.	Benzo[k]fluoranthene	C20H12	252.3	6.4
28.	3-Methylcholanthrene	C21H16	268.3	6.4
29.	Dibenz[a,h]anthracene	C22H14	278.3	6.5
30.	Dibenz[a,j]anthracene	C22H14	278.3	6.5
31.	Benzo[g,h,i]perylene	C22H12	276.3	6.6

32.	Benzo[b]triphenylene	C22H14	323.3	6.7
33.	Pentacene	C22H14	278.3	6.7
34.	Benzo[b]perylene	C24H14	302.3	7.0
35.	Dibenzo[a,h]pyrene	C24H14	302.3	7.0
36.	Indeno[1,2,3-c,d]pyrene	C22H12	276.3	7.0
37.	Dibenzo[a,l]pyrene	C24H14	302.3	7.2
38.	Coronene	C24H12	300.3	7.2
39.	Dibenzo[a,i]pyrene	C24H14	302.3	7.3
40.	Dibenzo[a,e]pyrene	C24H14	302.3	7.3
41.	Dibenzo[g,p]chrysene	C26H16	328.4	7.4
42.	Dibenzo[a,j]perylene	C28H16	352.4	8.3
43.	Dibenzo[a,g]coronene	C32H16	400.4	8.4
44.	Dibenzo[bc,kl]coronene	C30H14	374.4	9.2
45.	Ovalene	C32H14	398.4	10.0

*—Data were selected from PubChem database [57].

Table S3. Calculated partition coefficients of some persistent organic pollutants *.

No.	Compound	Molecular formula	Molecular weight	Xlog P3-AA
1.	Endrin	C12H8Cl6O	380.8	3.7
2.	Dieldrin	C12H8Cl6O	380.8	3.7
3.	Heptachlor	C10H5Cl7	373.3	4.3
4.	Chlordane	C10H6Cl8	409.7	4.9
5.	Pentachlorobenzene	C6HCl5	250.3	5.2
6.	Mirex	C10Cl12	545.5	5.3
7.	2,2',5-Trichlorobiphenyl	C12H7Cl3	257.5	5.6
8.	2,4,4'-Trichlorobiphenyl	C12H7Cl3	257.5	5.6
9.	Hexachlorobenzene	C6Cl6	284.7	5.7
10.	2,4',5-Trichlorobiphenyl	C12H7Cl3	257.5	5.8
11.	2,3',5-Trichlorobiphenyl	C12H7Cl3	257.5	5.8
12.	2,3,4-Trichlorobiphenyl	C12H7Cl3	257.5	5.9
13.	2,5,2',5'-Tetrachlorobiphenyl	C12H6Cl4	291.9	6.1
14.	3,3',5,5'-Tetrachlorobiphenyl	C12H6Cl4	291.9	6.1
15.	Toxaphene Parlar 44	C10H10Cl8	413.8	6.1
16.	p,p'-DDD	C14H10Cl4	320.0	6.2

17.	2,2',4,4'-Tetrachlorobiphenyl	C12H6Cl4	291.9	6.3
18.	2,3',4,4'-Tetrachlorobiphenyl	C12H6Cl4	291.9	6.3
19.	1,3,6,8-Tetrachlorodibenzo[p]dioxin	C12H4Cl4O2	321.9	6.3
20.	2,3,7,8-Tetrachlorodibenzo[p]dioxin	C12H4Cl4O2	321.9	6.4
21.	1,2,3,7,9-Pentachlorodibenzo[p]dioxin	C12H3Cl5O2	356.4	6.5
22.	2,3,7,8-Tetrachlorodibenzofuran	C12H4Cl4O	305.9	6.5
23.	3,3',4,4'-Tetrachlorobiphenyl	C12H6Cl4	291.9	6.6
24.	2,3,3',4,4'-Pentachlorobiphenyl	C12H5Cl5	326.4	6.6
25.	1,2,3,7,8-Pentachlorodibenzo[p]dioxin	C12H3Cl5O2	356.4	6.6
26.	2,3,4,5,6-Pentachlorobiphenyl	C12H5Cl5	326.4	6.7
27.	1,2,3,6,7-Pentachlorodibenzo[p]dioxin	C12H3Cl5O2	356.4	6.7
28.	o,p'-DDT	C14H9Cl5	354.4	6.7
29.	p,p'-DDT	C14H9Cl5	354.4	6.9
30.	1,2,3,4,5,6-Hexachloronaphthalene	C10H2Cl6	334.8	6.9
31.	1,2,3,6,7,8-Hexachloronaphthalene	C10H2Cl6	334.8	6.9
32.	1,2,4,5,6,8-Hexachloronaphthalene	C10H2Cl6	334.8	6.9
33.	1,2,3,6,7,8-Hexachlorodibenzofuran	C12H2Cl6O	374.8	6.9
34.	p,p'-DDE	C14H8Cl4	318.0	7.0
35.	1,2,4,6,7,9-Hexachlorodibenzofuran	C12H2Cl6O	374.8	7.0
36.	1,2,3,5,6,7-Hexachloronaphthalene	C10H2Cl6	334.8	7.0
37.	1,2,3,4,6,7-Hexachloronaphthalene	C10H2Cl6	334.8	7.0
38.	2,4,5,3',4'-Pentabromobiphenyl	C12H5Br5	548.6	7.0
39.	1,2,3,6,7,9-Hexachlorodibenzofuran	C12H2Cl6O	374.8	7.1
40.	2,3,4,6,7,8-Hexachlorodibenzofuran	C12H2Cl6O	374.8	7.1
41.	2,3',4,4',5-Pentachlorobiphenyl	C12H5Cl5	326.4	7.1
42.	3,3',4,4',5-Pentachlorobiphenyl	C12H5Cl5	326.4	7.3
43.	1,2,3,7,8,9-Hexachlorodibenzofuran	C12H2Cl6O	374.8	7.4
44.	1,2,3,4,7,8,9-Heptachlorodibenzofuran	C12HCl7O	409.2	7.5
45.	1,2,3,4,6,7,9-Heptachlorodibenzofuran	C12HCl7O	409.2	7.6
46.	1,2,3,4,5,6,8-Heptachloronaphthalene	C10HCl7	369.2	7.6
47.	1,2,3,4,5,6,7-Heptachloronaphthalene	C10HCl7	369.2	7.6
48.	1,2,3,4,7-Pentachlorodibenzo[p]dioxin	C12H3Cl5O2	356.4	7.8
49.	1,2,3,4,6,7,8-Heptachlorodibenzofuran	C12HCl7O	409.2	7.9
50.	Octachlorodibenzo[p]dioxin	C12Cl8O2	459.7	8.1

51.	Octachloronaphthalene	C10Cl8	403.7	8.2
52.	2,2',3,3',4,5',6-Heptabromodiphenyl ether	C12H3Br7O	722.5	8.3
53.	Octachlorodibenzofuran	C12Cl8O	443.7	8.5
54.	2,2',3,3',4,4',5,5'-Octabromodiphenyl ether	C12H2Br8O	801.4	9.0
55.	4-Fluoro-2,2,3,3,4,5,5,6,6-nonabromodiphenyl ether	C12Br9FO	898.3	9.8

*—Data were selected from PubChem database [57].
